# How do publicly procured school meals programmes in sub-Saharan Africa improve nutritional outcomes for children and adolescents: a mixed-methods systematic review

**DOI:** 10.1017/S1368980024001939

**Published:** 2024-10-18

**Authors:** Julia Liguori, Hibbah Araba Osei-Kwasi, Mathilde Savy, Silver Nanema, Amos Laar, Michelle Holdsworth

**Affiliations:** 1 UMR MoISA (Montpellier Interdisciplinary Centre on Sustainable Agri-Food Systems), University of Montpellier, CIRAD, CIHEAM-IAMM, INRAE, Institut Agro Montpellier, IRD, Montpellier 34000, France; 2 School of Sport, Exercise and Health Sciences, Loughborough University, Loughborough, UK; 3 University of Ghana, Department of Population, Family & Reproductive Health, School of Public Health, Accra, Ghana

**Keywords:** Public procurement, School meals, Food environment, Policies, Sub-Saharan Africa

## Abstract

**Objective:**

This review aimed to (i) synthesise evidence of the impact of publicly procured school meals programmes on nutritional outcomes of children/adolescents (5–18 years) in sub-Saharan Africa and (ii) identify challenges and facilitators to implementing effective school meals programmes.

**Design:**

Mixed-methods systematic review (*n* 7 databases). Nutritional outcomes assessed were anthropometrics (underweight, stunting, wasting, overweight/obesity), micronutrient deficiencies, food consumed and food environment. Qualitative findings were coded using a nine-step school food system framework: *production of food, wholesale and trading, transportation and storage, processing and distribution, food preparation, distribution to students, student stakeholders, community involvement* and *infrastructure support*.

**Setting:**

Sub-Saharan Africa.

**Participants:**

Children/adolescents (5–18 years), parents, school personnel and government officials.

**Results:**

Thirty-three studies (twenty-six qualitative, seven quantitative) from nine sub-Saharan African countries were included. Six studies found a positive impact of publicly procured school meals programmes on nutritional outcomes (wasting (*n* 1), stunting (*n* 3), underweight (*n* 1), vitamin A intake (*n* 1) and dietary diversity (*n* 1)). Fifty-three implementation challenges were identified, particularly during *food preparation* (e.g. training, payment), *distribution to students* (e.g. meal quantity/quality/diversity, utensils) and *infrastructure support* (e.g. funding, monitoring, coordination). Implementation facilitators were identified (*n* 37) across *processing and distribution* (e.g. programme coordination), *student stakeholders* (e.g. food preferences, reduced stigma) and *community involvement* (e.g. engagement, positive perceptions). Included policy recommendations targeted *wholesale and trading*, *food preparation*, *student stakeholders* and *infrastructure support* in nine, fifteen and twenty-five studies, respectively.

**Conclusions:**

As many challenges remain, strengthening implementation (and therefore the nutritional impact) of school meals programmes in sub-Saharan Africa requires bold commitment and improved coordination at multiple levels of governance.

Growing global interest in national school meals programmes (SMP) centres around school meals as a panacea for educational and nutritional outcomes^(1)^. SMP provide breakfast, lunch, snacks and/or take-home rations to students to improve enrolment, attendance, nutritional status and gender-based food allocation practices^(2)^. Approximately 65·4 million primary school children participated in SMP spanning fifty-one African countries in 2021^(3)^. Despite progress, school meal coverage remains the lowest on the African continent, with an estimated 73 % of the world’s most vulnerable children missed^(4)^.

SMP at the national level can use public procurement (i.e. public purchase of goods from the private sector) as an opportunity to include healthy food purchasing guidelines to promote food systems change across sub-Saharan Africa (SSA). In order for public procurement to succeed, a shift in government practices to procure food with economic, environmental and social benefits is prerequisite, with some studies suggesting that political will has already shifted^(5,6)^. In SSA, this can be seen with the surge of countries investing in nationally funded SMP, including Angola, Benin, Burkina Faso, Burundi, Cameroon, Central African Republic, Chad, Côte d’Ivoire, Democratic Republic of the Congo, Ethiopia, Gambia, Guinea, Kenya, Lesotho, Liberia, Madagascar, Mali, Mauritania, Mozambique, Namibia, Niger, Nigeria, Congo, Rwanda, Senegal, Sierra Leone, Somalia, South Africa, South Sudan, Sudan, Tanzania, Togo, Uganda, Zambia and Zimbabwe^(7)^, often representing large proportions of government budgets. The introduction of guidelines to regulate what foods are served and sold in and around schools^(2,8,9)^ can include criteria for not only what food schools should purchase (e.g. local, nutritious, culturally acceptable) but also from whom the food is sourced (e.g. smallholder farmers, female farmers, small cooperatives), thus extending potential benefits to students, smallholder farmers and local communities in both centralised and decentralised procurement models^(2,5,10,11)^. Home-grown school feeding embodies this goal, shifting the focus to context-specific approaches for food procurement and incorporating national and pan-African guidelines to increase local food sourcing^(2,12)^. It also underscores increased understanding that SMP need to adapt as programmatic needs evolve and ensure that regional demand for local food sourcing, freshness and taste preferences are met.

Beyond SMP, a healthy school food environment (i.e. ‘*all the spaces, infrastructure and conditions inside and around school premises where food is available, obtained, purchased and/or consumed*’)^(13)^ can also act as a driver to reduce diet-related non-communicable diseases, alongside persistent undernutrition in children and adolescents in SSA^(13,14)^. While childhood and adolescence represent two key life stages for growth and development^(15)^, evidence on the impact of school meals on nutritional outcomes in SSA is limited^(16)^.

Evidence from high-income countries demonstrates that implementing criteria for nutritious food to publicly procured SMP will improve the nutritional quality of food consumed among children^(17)^. However, the true potential of procurement as a driver of change in schools remains unknown as few countries in SSA have implemented these models and/or have monitoring and evaluation mechanisms^(2,6,10)^. To the best of our knowledge, this review is the first to look at national SMP and food procurement policies as a way to improve nutritional outcomes among children and adolescents and to shape the food environment in SSA. This review aimed to (1) synthesise the evidence of the impact of publicly procured SMP on school food environments and nutritional outcomes of children and adolescents (5–18 years) in SSA and (2) identify the challenges and facilitators to implementing effective SMP.

## Methods

### Reporting

A systematic review was conducted following the Preferred Reporting Items for Systematic Reviews and Meta-Analyses (PRISMA) guidelines^(18)^. The protocol for this review was registered with the International Prospective Register of Systematic Reviews (PROSPERO 2022 CRD42022354440).

### Eligibility criteria

The Population Intervention Comparison Outcome and Study type (PICOS) model was used: *Population* (children and adolescents in primary/secondary schools); *Intervention* (publicly procured SMP (i.e. nationally funded government programme)); *Context* (in SSA); *Outcomes* (anthropometrics (underweight, stunting, wasting, overweight/obesity), micronutrient deficiencies, food consumption and food environment) or (challenges and facilitators to programme implementation); and *Study type* (randomised and non-randomised controlled trials, quasi-randomised trials, prospective cohort studies with repeated cross-sectional design, qualitative studies, mixed-method studies). Studies conducted over the past 10 years in English and French were eligible for inclusion. All eligibility criteria are included in online supplementary material, Supplemental Material 1.

### Search strategy and data extraction

Scoping searches were conducted and checked by a reference librarian to identify relevant studies. The search syntax was developed in PubMed and then adapted to meet database-specific requirements (e.g. Medical Subject Headings). Searches were conducted in September 2022 in seven databases: PubMed (MEDLINE), Cochrane Central Register of Controlled Trials (CENTRAL), CINAHL (EBSCO), EMBASE, Google Scholar, e-Library of Evidence for Nutrition Actions (ELENA) and Global Database on Implementation of Nutrition Action (GINA). Grey literature was also included. At both the title/abstract and the full-text screening stages, 15 % of excluded articles were reviewed by a second reviewer (HOK, JL, SN) to ensure inter-rater accuracy among excluded articles. Reference lists of background articles, systematic reviews and included studies were hand searched in March 2023 for additional references. Additionally, the ‘cited by’ function in Google Scholar was used as a snowball technique to identify relevant articles.

### Data extraction

*Google Forms* was used for data extraction. HOK, JL, MH and MS piloted and conducted the data extraction, including information on study design, study setting (country, rural/urban, primary/secondary school), participant characteristics (age, sample size, role in school) and type of intervention (school meal type, duration, period of evaluation, cost). Additional information was extracted on nutritional outcomes measured (i.e. anthropometrics, micronutrient deficiencies, food consumed or food environment), implementation challenges and/or facilitators and author-based policy recommendations (see online supplementary material, Supplemental Material 2).

### Quality appraisal

Included studies were independently appraised twice (JL, HOK, MH, MS) using the Mixed Methods Appraisal Tool (MMAT)^(19)^. This tool was designed to critically appraise multiple types of research methodologies in systematic reviews^(19,20)^. As Cochrane guidelines advise appraisers to judge the quality of evidence, without giving a definitive score^(21)^, each article was given a colour (red = low, amber = medium, green = high) to indicate overall quality. Any disagreement between reviewers was resolved by discussion.

### Framework for analysis

**Fig. 1:**
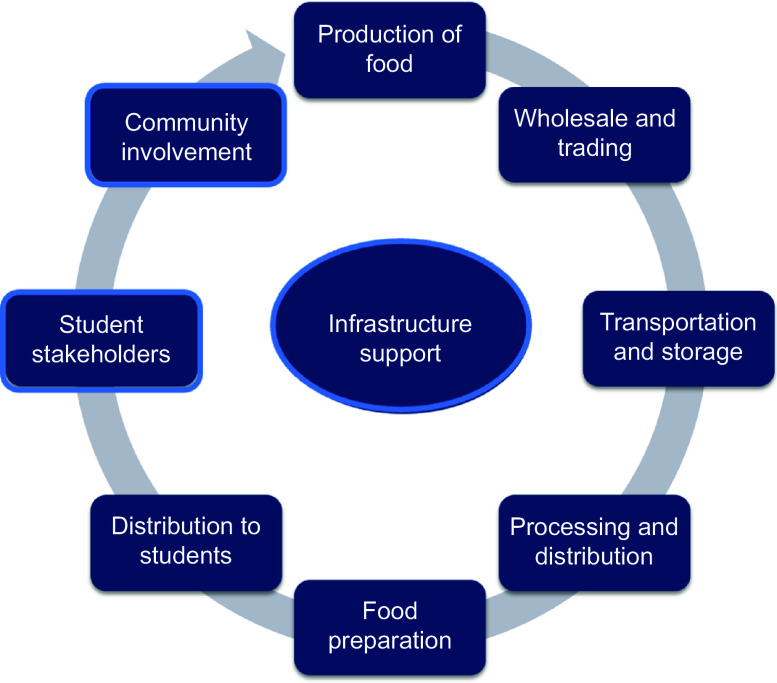
School food system framework adapted from Drake *et al.*, 2016; De Carvalho *et al.*, 2011; Moepeng, 2016; WFP, 2018; and WHO, 2021

A school food system framework (Fig. 1) was developed, integrating concepts from others^(1,14,22–24)^. The framework details each step of the school food system: *production of food, wholesale and trading, transportation and storage, processing and distribution, food preparation, distribution to students, student stakeholder, community involvement and infrastructure support*. This framework differs from prior models as it includes an additional step to include *students* as active stakeholders within the SMP and a stand-alone step for *community involvement*. In addition, *infrastructure support*, adapted from Food-Epi domains^(25)^, was added as a cross-cutting category encompassing leadership, governance, monitoring and evaluation, funding, resource platforms for interaction and health in all policies.

### Data synthesis

Due to the small number and methodological heterogeneity of quantitative studies, data were synthesised descriptively and reported by nutritional outcome. For qualitative findings, a thematic analysis of barriers and facilitators was conducted. Themes were identified from the analysis of extracted text and coded in NVivo12^(26)^. Data were synthesised with a framework matrix including nodes for different themes/subthemes^(27)^. Nodes were then broken down into nine steps, representing the different steps of the school food system. Enhancing transparency in reporting the synthesis of qualitative research (ENTREQ) statement was followed^(28)^. Author-based policy recommendations emerging from included studies were also mapped across the school food system framework.

## Results

### Description of included studies

**Fig. 2:**
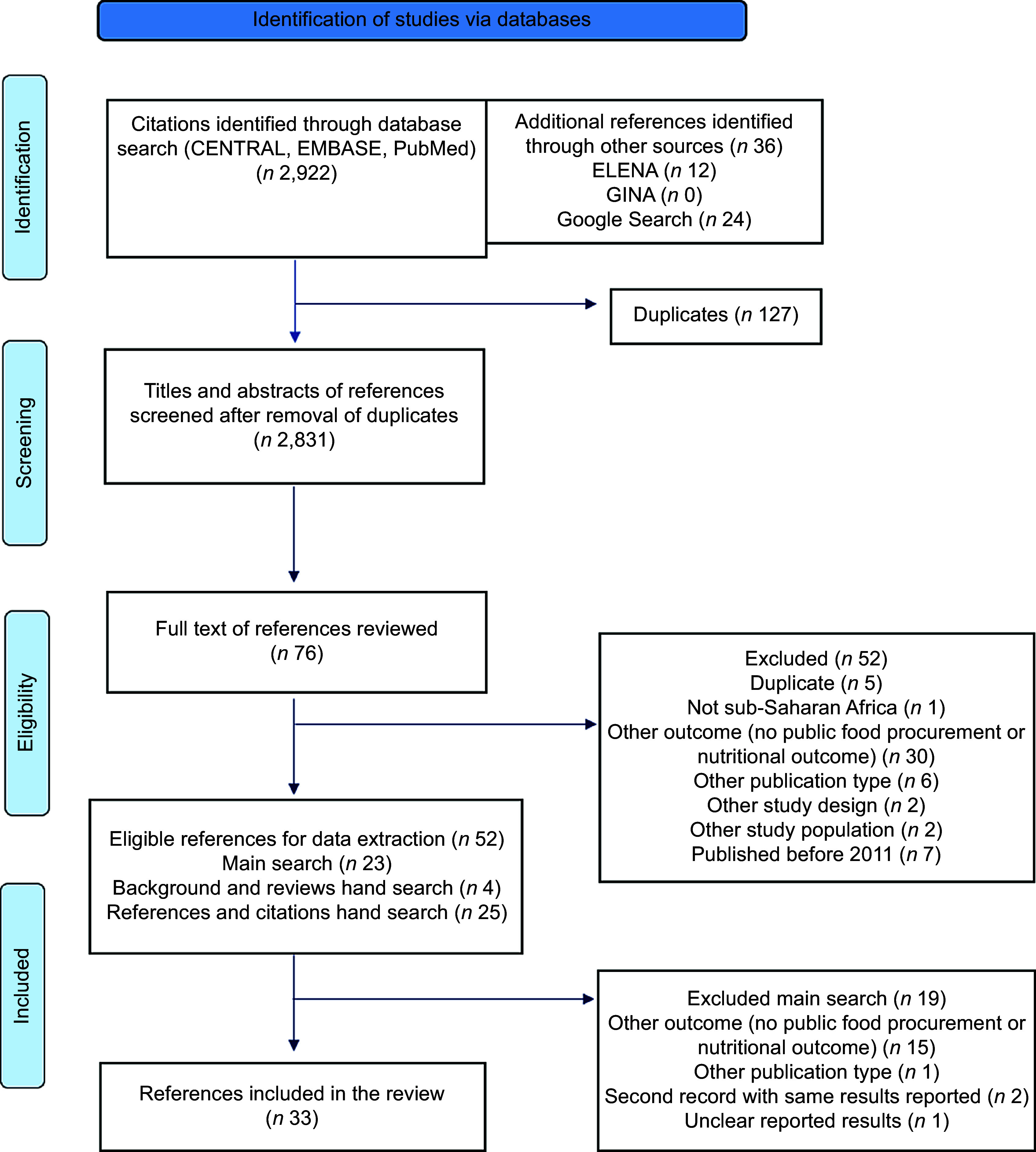
PRISMA diagram detailing the screening process

**Fig. 3:**
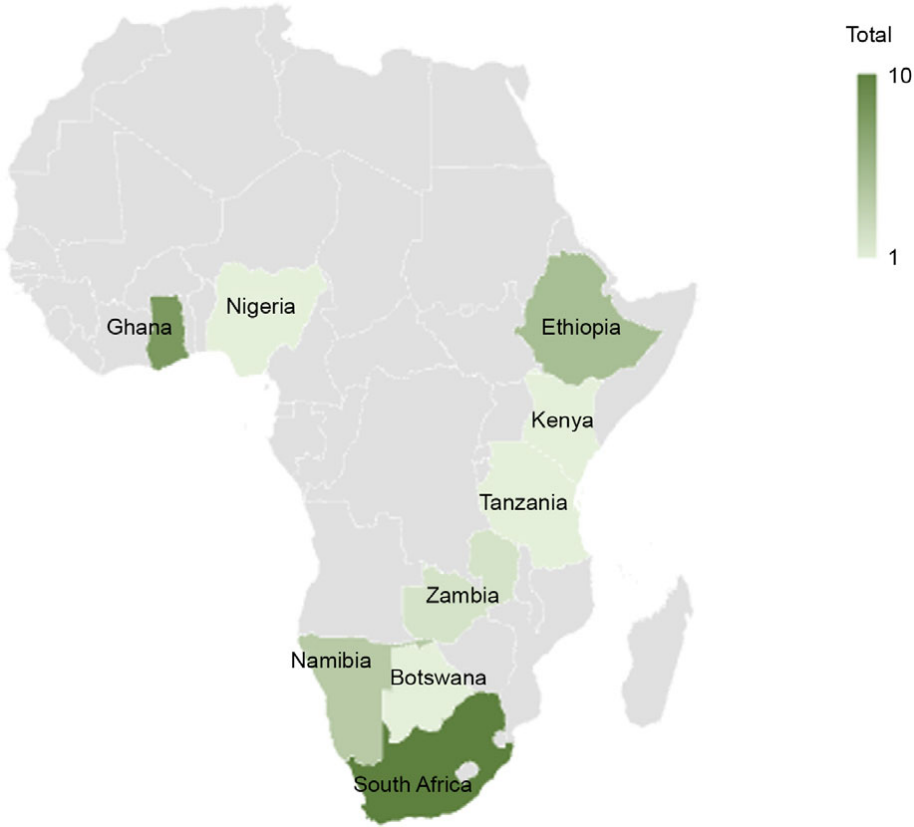
Map showing the distribution of research of publicly procured school meals programmes and nutritional outcomes in sub-Saharan Africa

In total, thirty-three studies were included in this review (Fig. 2) in nine of the forty-six SSA countries (Fig. 3). Most studies were conducted in either South Africa (*n* 10) or Ghana (*n* 9), followed by Ethiopia (*n* 4), Namibia (*n* 4) and Zambia (*n* 2). Only one study was identified in each of Botswana, Kenya, Nigeria and Tanzania. All nine countries reported having national SMP. Most studies focused on primary schools (*n* 27), while four studies^(29–32)^ included secondary schools, and three studies^(33–35)^ included both. The thirty-three studies comprised eighteen journal articles, ten master-level theses, three international reports, two doctoral theses and one working paper. Excluded studies at the data extraction stage are available (see online supplementary material, Supplemental Material 3).

Seven studies elucidated the first research objective: to synthesise the evidence of the impact of publicly procured SMP on nutritional outcomes and school food environments of children/adolescents (5–18 years) in SSA (Table 1). All seven studies used quantitative methods, including two randomised control trials^(36,37)^, two longitudinal cohorts with multiple points of cross-sectional data collection^(38,39)^, two single collect cross-sectional studies^(29,40)^ and one non-randomised trial^(41)^, spanning Ethiopia, Ghana, Kenya, Nigeria and South Africa.

**Table 1:** Characteristics of studies assessing the impact of procured school meals programmes (SMP) on nutritional outcomes

Author	Study design	Country	Zone	Intervention (modality)	Duration of intervention	Number of schools (sampling)	Participants (sampling)	Student age	Reported outcomes	Key findings	Quality Appraisal MMAT Colour Score
Abizari *et al.*, 2021^(29)^	Cross-sectional	Ghana	Urban	School meal (lunch)	1 month	3 (purposive)	Secondary school students (*n* 403). Random.	12–17 years	Dietary outcomes (dietary diversity score (DDS) 24 h recall; 72 h recall)	○ Over a 3-day lunch comparison, SMP beneficiaries had a 1-unit increase in DDS (7·5 *v*. 6·5 food groups, *P* < 0·001) compared with non-beneficiaries, with significant differences observed for white roots and tubers, eggs, legumes/nuts/seeds and oils/fat. ○ In comparing whole-day meals, a 2-unit increase in DDS of whole-day meals of beneficiary students compared with non-beneficiaries (11·5 *v*. 9·3; *P* < 0·001) for dark green leafy vegetables, vitamin A-rich fruits, organ meat/flesh meat, fish/seafood, legumes/nuts/seeds and milk/milk products. However, the school meal rarely served fruit, vegetables, meat and dairy products.	Amber
Desalegn *et al.*, 2022^(38)^	Cohort	Ethiopia	Rural	School meal (lunch)	12 months	16 (random)	Primary school students (*n* 463). Random.	10–14 years	Anthropometric outcomes (BAZ; HAZ); Micronutrient deficiencies outcome (anaemia, Hb concentration)	○ No significant effect on overall anthropometric status (HAZ, BAZ). ○ No significant difference on overall Hb concentration	Amber
Faber *et al.*, 2013^(40)^	Cross-sectional	South Africa	Urban; peri-urban; rural	School meal (not reported)	7 months	90 (purposive)	School principal (*n* 85), programme coordinator (*n* 77), food handlers (*n* 84). Sample method not reported.	11–13 years	Food environment outcome (food provision at school level)	○ School policy on food sold (clean and healthy) to the learners was only implemented in 19 % of schools. ○ School policy on foods brought to school, focusing on healthy foods (fruit) and limiting ‘junk’ food in the lunch box, was found in 13 % of schools. ○ Most schools did not comply with the required daily serving of vegetables and/or fruit. ○ 64–87 % of food handlers received training on hygiene, storage and portion sizes (food handlers).	Red
Gelli *et al.*, 2019^(36)^	Cluster RCT	Ghana	Urban; rural	School meal (lunch)	34 months	91 (random)	Primary school students (*n* 3170). Random. Food handlers (*n* 55). Purposive.	5–15 years	Anthropometric outcomes (BAZ; HAZ) *	○ School meals had no effect on HAZ and BAZ in children aged 5–15 years. ○ In subgroup analysis, the SMP intervention improved HAZ, in children in households living below the poverty line (effect size: 0·22 sd) and in girls living in the northern regions (effect size: 0·12 sd).	Green
Neerrvoort *et al.*, 2013^(41)^	Non-randomised trial	Kenya	Urban slum	School meal (lunch, snack); supplementation (vitamin A, iron); Nutrition education	12 months school meal; 3 months vitamin supplementation	1 (not reported)	Primary school students (*n* 148). Convenience.	2–10 years	Anthropometric outcomes (stunting, underweight, wasting) *; micronutrient outcomes (anaemia, severe anaemia)	○ SMP beneficiaries were less stunted (12 *v*. 22 %, *P* = 0·02) and wasted (0 *v*. 11 %, *P* = 0·02) than those in the control group. However, no children were underweight (0 %) in the intervention group at baseline compared with 11 % of children being underweight in the control group at the same time, which could bias the sample. Data were also collected for the control group 1 year after the intervention group. ○ Prevalence of anaemia among SMP beneficiaries was lower than non-SMP beneficiaries (19 *v*. 42 %, *P* = 0·01). Severe anaemia was not reported in any group.	Red
Oyela *et al.*, 2023^(39)^	Cohort	Nigeria	Not reported	School meal provision (home-grown school feeding)	6 months	10 (random)	Primary school students (*n* 500). Purposive.	5–7 years	Anthropometric outcomes (stunting, underweight, wasting)	○ Improvement in the nutritional status of the beneficiary group compared with the non-beneficiary group, with a general improvement in the anthropometric measurements at 3 and 6 months, with more gains made at 6 months. However, at baseline, there were major differences in nutritional status between intervention and control groups: stunting 22 % *v*. 44·4 %; wasting 12 % *v*. 6 %; underweight 23·2 % *v*. 2·8 %. ○ Reported statistically significant improvements in underweight (*P* = 0·001, F = 23·847, η2 = 0·046) and stunting (*P* = 0·04, F = 4·083, η2 = 0·008) at 6 months (5 % variance in underweight and ∼1 % variance in stunting likely attributed to the SMP. ○ No statistically significant relationship between the SMP and wasting (*P* = 0·30, F = 1·075, η2 = 0·002) was observed. ○ In subgroup analysis, there was an improvement in the nutritional status of both male and female children at 6 months, with statistically significant gender differences were observed in underweight levels of both males and females at 3 months and 6 months (t1 = 2·378, p1 = 0·018; t2 = 2·123, p1 = 0·035), respectively, and a significant gender difference was observed in stunting level at 6 months (t = 2·152, *P* = 0·032).	Amber
Van der Hoeven *et al.*, 2015^(37)^	RCT	South Africa	Rural	School meal provision (lunch or lunch with green leafy vegetables)	3 months	2 (purposive)	Primary school students (*n* 167). Random.	6–12 years	Micronutrient outcomes (anaemia, Hb concentration; Serum ferritin; serum transferrin receptor; Zinc (serum Zn; serum retinol concentrations; Zn protoporphyrin)	○ No effect of adding African leafy vegetables to the SMP observed in anaemia, Zinc deficiency and Iron deficiency and serum retinol among the SMP beneficiary group. ○ The prevalence of subclinical Vitamin A deficiency decreased significantly in the intervention group from 7·0 % at baseline to 1·3 % at end point (*P* = 0·015), with no change in the control group.	Amber

BAZ, BMI-for-age Z-score; Hb, Haemoglobin; HAZ, height-for-age Z-score; RCT, randomised controlled trial; SMP, School Meal Programme.

* WHO standards used.

Twenty-six studies, using qualitative methods, shed light on the second research objective to identify challenges and facilitators to implementing effective procurement in SMP in SSA (Table 2). These studies were conducted in seven countries: Botswana, Ethiopia, Ghana, Namibia, South Africa, Tanzania and Zambia. There was some overlap between challenges and facilitators and was often context dependent (Table 3).

**Table 2:** Characteristics of studies identifying challenges and facilitators of school meal programme implementation

Author	Country	Zone	Number of schools (sampling)	Participants (sampling)	Student age	Data collection method	Implementation		
							Challenges	Facilitators	Quality Appraisal MMAT Score
Banda^†^, 2017^(42)^	Zambia	Not reported	15 (random)	Primary school children (*n* 15); parents (*n* 15); government official (*n* 1); teachers (*n* 9); head of school (*n* 6) (purposive)	Not reported	Individual interviews; semi-structured questionnaire	✓	✓	Green
Daitai^†^, 2017^(30)^	South Africa	Rural	1 (purposive)	Parents (*n* 5); food handlers (2); teachers (2); head of school (*n* 1); supervisor at circuit level (*n* 1); farmers (*n* 5); students (*n* 10) (quota)	17–18 years	Individual interviews; observation; document analysis	✓	✓	Green
Darko^†^, 2014^(46)^	Ghana	Urban; Rural	2 (purposive)	Student leadership; school leadership; parent–teacher association. Sample size not reported (random)	Not reported	Focus group	✓		Amber
Dei, 2014^(47)^	South Africa	Rural	1 (purposive)	Primary school students (*n* 112); teachers (*n* 9) (probability)	Grade 6–7 (∼11–13)	Focus group	✓	✓	Amber
Desalegn *et al.*, 2022^(43)^	Ethiopia	Rural	8 (random)	Primary school children; parents; government officials; teachers; head of school. Sample size not reported (sample method not reported)	10–14 years	Individual interviews; focus group	✓	✓	Green
Ellis, 2012^(57)^	Namibia	Urban; peri-urban; rural	15 (purposive)	Primary school children; parents; food handlers; head of school. Sample size not reported. Government officials (*n* 27) (sample method not reported)	Not reported	Individual interviews; focus group	✓	✓	Red
Fernandes *et al.*, 2016^(48)^	Ghana	Not reported	Not reported	Government officials; food handlers. Sample size not reported (sample method not reported)	Not reported	Focus group; monitoring reports; observation	✓		Amber
Fernandes *et al.*, 2017^(33)^	Ghana	Urban; rural	111 (purposive)	Parents (*n* 72) (random)	5–17 years	Focus group	✓	✓	Amber
Hamupembe^†^, 2016^(44)^	Namibia	Urban	2 (purposive)	Teachers (*n* 16) (random); head of school (*n* 2); Namibian School Feeding Programme focal person (*n* 2) (purposive)	Not reported	Focus group; observation	✓	✓	Green
Khama*, 2022^(49)^	Namibia	Not reported	2 (purposive)	Head of school (*n* 2); teachers (*n* 5), coordinators (*n* 4), school board members (*n* 2); service providers (*n* 1) (purposive)	Not reported	Individual interviews; focus group; observation	✓	✓	Amber
Langsford^†^, 2018^(50)^	South Africa	Urban	2 (purposive)	Primary school children; parents; government officials; food handlers; school management; teachers. Sample size not reported (purposive)	7–13 years	Individual interviews; observation	✓	✓	Amber
Mafugu, 2021^(31)^	South Africa	Not reported	5 (purposive)	One teacher coordinator (*n* 1), head of school (*n* 4), food service providers (*n* 7), food handlers (*n* 6), government officials (*n* 2) (purposive)	Grade 12 (∼17–18)	Individual interviews	✓	✓	Green
Mensah, 2019^(51)^	Ghana	Not reported	56 (not reported)	Food caterers (*n* 11); head of school (*n* 5); household respondent (*n* 5); Grain Banks Committee Representatives (not reported) (sample method not reported)	Not reported	Individual interviews; focus group	✓	✓	Amber
Mensah & Karriem, 2021^(34)^	South Africa	Rural	12 (random)	Government officials (not reported); teachers (*n* 12); head of school (*n* 12); food suppliers; farmers (*n* 43) (purposive)	Not reported	Individual interviews	✓	✓	Amber
Moepeng, 2016^(23)^	Botswana	Urban; Peri-urban; Rural	4 (purposive)	Primary school children; parents; kitchen staff; teachers; parent–teacher association members, community members; farmers; food suppliers. Sample size not reported (purposive)	Not reported	Individual interviews; focus group; observation	✓	✓	Green
Molotja*, 2019^(45)^	South Africa	Rural	11 (random)	Primary school children (not reported). Random; food handlers (*n* 30). Purposively; programme officers (*n* 5); teachers (*n* 14) (sample method not reported)	10–15 years	Individual interviews; focus group; participatory rural appraisal (observations, note taking, photographs, Venn diagrams, seasonal calendars)	✓	✓	Green
Okae-Adjei *et al.*, 2016^(52)^	Ghana	Not reported	10 (random)	Primary school children; parents. Sample sizes not reported. Random; government officials. Sample size not reported (purposive)	Not reported	Individual interviews; observation	✓	✓	Amber
Rector *et al.*, 2021^(32)^	Tanzania	Rural	10 (not reported)	Secondary school children; parents; government officials; head of school; biology teachers. Sample size not reported (sample method not reported)	14–15 years	Individual interviews; focus group	✓	✓	Amber
Rendall-Mkosi *et al.*, 2013^(35)^	South Africa	Urban; rural	12 (purposive)	Parents; government officials; kitchen staff; teachers; head of school; food handlers; food suppliers. Sample size not reported (purposive)	Not reported	Individual interviews; focus group; observation	✓	✓	Amber
Sanousi, 2019^(53)^	South Africa	Urban	4 (not reported)	Government officials (*n* 1); Head of school (*n* 4); teacher coordinators (*n* 4), food handlers (*n* 4); members of the school governing body (*n* 4) (purposive)	Not reported	Individual interviews	✓	✓	Amber
Sibanda^†^, 2012^(58)^	Namibia	Urban	1 (random)	Parents (*n* 8) (convenience)	11–16 years	Focus group	✓	✓	Red
Sichala^†^, 2020^(54)^	Zambia	Urban; rural	4 (purposive)	Learners (not reported), school heads, programme focal teachers, parents, key community leaders, government representative, NGO representative (purposive)	Not reported	Individual interviews; focus group; observation; quantitative questionnaire	✓	✓	Amber
Sulemana *et al.*, 2013^(59)^	Ghana	Urban; peri-urban; rural	17 (purposive)	Primary school children; food handlers; teachers; head of school; health workers; farmers; community members; programme officers. Sample size not reported (sample method not reported)	Not reported	Individual interviews; Focus Group	✓	✓	Red
Xie & Brownell, 2020^(55)^	Ethiopia	Urban	Not reported	Community stakeholders (*n* 7) (convenience)	Grade 1–8 (∼7–14)	Individual interviews	✓	✓	Amber
Yendaw & Dayour, 2015^(56)^	Ghana	Rural	1 (not reported)	Parents (*n* 155); head of school (*n* 1); food suppliers (*n* 1); assembly member (*n* 1); government officials (not reported) (sample method not reported)	Not reported	Individual interviews; observation	✓		Amber
Zenebe *et al.*, 2018^(60)^	Ethiopia	Not reported	6 (purposive)	FAO/WFP school meal programme coordinator (*n* 1); head of school (*n* 6); government officials (not reported) (purposive)	10–14 years	Individual interviews	✓	✓	Red

*PhD thesis; †master’s thesis.

**Table 3:** Challenges and facilitators to implementing publicly procured school meal programmes (SMP)

School food system step	Challenges (no of studies)	Studies	Facilitators (no of studies)	Studies
**Production of food**	3		2	
Local food production	3	Desalegn *et al.*, 2022^(43)^; Mensah & Karriem, 2021^(34)^; Moepeng 2013^(23)^	2	Ellis, 2012^(57)^; Moepeng, 2013^(23)^
**Wholesale and trading**	13		9	
Local procurement and home-grown school feeding programmes	5	Daitai *et al.*, 2018^(30)^; Ellis, 2012^(57)^; Moepeng, 2013^(23)^; Molotja, 2019^(45)^; Rendall-Mkosi *et al.*, 2013^(35)^	5	Desalegn *et al.*, 2022^(43)^; Mensah, 2019^(51)^; Mensah & Karriem, 2021^(34)^; Moepeng, 2013^(23)^; Rendall-Mkosi *et al.*, 2013^(35)^
Financial constraints and transparency	4	Mafugu, 2021^(31)^; Mensah & Karriem, 2021^(34)^; Xie & Brownell, 2020^(55)^; Zenebe *et al.*, 2018^(60)^	1	Rector *et al.*, 2021^(32)^
Quantity of food purchased	4	Banda, 2017^(42)^; Mafugu, 2021^(31)^; Mensah & Karriem, 2021^(34)^; Khama, 2022^(49)^	0	
Decentralised food procurement	2	Khama, 2022^(49)^; Langsford, 2018^(50)^	3	Langsford, 2018^(50)^; Mensah & Karriem, 2021^(34)^; Rendall-Mkosi *et al.*, 2013^(35)^
Centralised food procurement	1	Banda, 2017^(42)^	3	Moepeng, 2013^(23)^; Molotja, 2019^(45)^; Rendall-Mkosi *et al.*, 2013^(35)^
Reliability of suppliers (e.g. contracts, avoid conflict)	2	Khama, 2022^(49)^; Moepeng 2013^(23)^	3	Dei, 2014^(47)^; Mafugu, 2021^(31)^; Molotja, 2019^(45)^
Food procurement procedure in place (or lack thereof)	2	Mafugu, 2021^(31)^; Rector *et al.*, 2021^(32)^	1	Dei, 2014^(47)^
**Transportation and storage**	13		2	
On-time food deliveries	10	Banda, 2017^(42)^; Daitai *et al.*, 2018^(30)^; Dei, 2014^(47)^; Desalegn *et al.*, 2022^(43)^; Langsford, 2018^(50)^; Mensah & Karriem, 2021^(34)^; Moepeng, 2013^(23)^; Molotja, 2019^(45)^; Sanousi, 2019^(53)^; Sibanda, 2012^(58)^	1	Hamupembe, 2016^(44)^
Quality of food delivered (spoilage)	4	Desalegn *et al.*, 2022^(43)^; Hamupembe, 2016^(45)^; Moepeng, 2013^(23)^; Molotja, 2019^(45)^	0	
Infrastructure of food transportation and supply chain	3	Khama, 2022^(49)^; Moepeng, 2013^(23)^; Zenebe *et al.*, 2018^(60)^	0	
Quantity of food delivered	0		1	Sichala, 2020^(54)^
**Processing and distribution**	14		11	
Storage condition on-site	12	Banda, 2017^(42)^; Daitai *et al.*, 2018^(30)^; Dei, 2014^(47)^; Desalegn *et al.*, 2022^(43)^; Ellis, 2012^(57)^; Hamupembe, 2016^(44)^; Khama, 2022^(49)^; Mafugu, 2021^(31)^; Molotja, 2019^(45)^; Rendall-Mkosi *et al.*, 2013^(35)^; Xie & Brownell, 2020^(55)^; Zenebe *et al.*, 2018^(60)^	1	Hamupembe, 2016^(44)^
School gardens creation and maintenance	5	Banda, 2017^(42)^; Daitai *et al.*, 2018^(30)^; Ellis, 2012^(57)^; Rector *et al.*, 2021^(32)^; Sanousi, 2019^(53)^	3	Molotja, 2019^(45)^; Rector *et al.*, 2021^(32)^; Rendall-Mkosi *et al.*, 2013^(35)^
Programme coordination at all levels	0		10	Daitai *et al.*, 2018^(30)^; Dei, 2014^(47)^; Hamupembe, 2016^(44)^; Khama, 2022^(49)^; Langsford, 2018^(50)^; Mensah & Karriem, 2021^(34)^; Moepeng, 2013^(23)^; Molotja, 2019^(45)^; Rector *et al.*, 2021^(32)^; Sanousi, 2019^(53)^
**Food preparation**	17		9	
Unpaid (including delayed payments) and/or untrained food handlers	13	Banda, 2017^(42)^; Dei, 2014^(47)^; Desalegn *et al.*, 2022^(43)^; Ellis, 2012^(57)^; Hamupembe, 2016^(44)^; Khama, 2022^(49)^; Langsford, 2018^(50)^; Mafugu, 2021^(31)^; Mensah, 2019^(51)^; Molotja, 2019^(45)^; Okae-Adjei *et al.*, 2016^(52)^; Rendall-Mkosi *et al.*, 2013^(35)^	0	
Infrastructure to prepare school meal	11	Desalegn *et al.*, 2022^(43)^; Ellis, 2012^(57)^; Hamupembe, 2016^(44)^; Khama, 2022^(49)^; Langsford, 2018^(50)^; Mafugu, 2021^(31)^; Okae-Adjei *et al.*, 2016^(52)^; Rector *et al.*, 2021^(32)^; Rendall-Mkosi *et al.*, 2013^(35)^; Sulemana *et al.*, 2013^(59)^; Xie & Brownell, 2020^(55)^	5	Dei, 2014^(47)^, Hamupembe, 2016^(44)^, Langsford, 2018^(50)^, Rendall-Mkosi *et al.*, 2013^(35)^, Xie & Brownell, 2020^(55)^
Food safety measures and training	9	Banda, 2017^(42)^; Hamupembe, 2016^(44)^; Khama, 2022^(49)^; Mafugu, 2021^(31)^; Molotja, 2019^(45)^; Okae-Adjei *et al.*, 2021^(32)^; Xie & Brownell, 2020^(55)^; Zenebe *et al.*, 2018^(60)^	5	Banda, 2017^(42)^, Daitai *et al.*, 2018^(30)^, Moepeng, 2013^(23)^, Rendall-Mkosi *et al.*, 2013^(35)^, Xie & Brownell, 2020^(55)^
Tools for measurements and consistency	3	Ellis, 2012^(57)^; Hampuembe, 2016^(44)^; Sibanda, 2012^(58)^	1	Fernandes *et al.*, 2017^(33)^
Safety standards in kitchen	2	Langsford, 2018^(50)^; Rendall-Mkosi *et al.*, 2013^(35)^	0	
Lack of guidelines for meal preparation	1	Hamupembe, 2016^(44)^	0	
Lack of nutrition knowledge of food handlers	1	Ellis, 2012^(57)^	0	
Food not meeting SMP requirements	1	Langsford, 2018^(50)^	0	
Timing of food on menus to avoid spoilage	1	Rendall-Mkosi *et al.*, 2013 ^(35)^	0	
**Distribution to students**	20		5	
Meal quality and quantity (dietary diversity)	13	Daitai *et al.*, 2018^(30)^; Darko, 2014^(56)^; Dei, 2014^(47)^; Desalegn *et al.*, 2022^(43)^; Ellis, 2012^(57)^; Khama, 2022^(49)^; Moepeng, 2013^(23)^; Molotja, 2019^(45)^; Okae-Adjei *et al.*, 2016^(52)^; Rendall-Mkosi *et al.*, 2013^(35)^; Sanousi, 2019^(53)^; Sichala, 2020^(54)^; Suelmana *et al.*, 2013^(59)^	0	
Supplies of cutlery and serving equipment not provided/provided	9	Banda, 2017^(42)^; Dei, 2014^(47)^; Desalegn *et al.*, 2022^(43)^; Ellis, 2012^(57)^; Hamupembe, 2016^(44)^; Khama, 2022^(49)^; Molotja, 2019^(45)^; Sulemana *et al.*, 2013^(59)^	1	Ellis, 2012^(57)^
Time constraints	9	Banda, 2017^(42)^; Dei, 2014^(47)^; Ellis, 2012^(57)^; Hamupembe, 2016^(44)^; Khama, 2022^(49)^; Mafugu, 2021^(31)^; Rendall-Mkosi *et al.*, 2013^(35)^; Sulemana *et al.*, 2013^(59)^; Zenebe *et al.*, 2018^(60)^	0	
Infrastructure in/around dining space	6	Khama, 2022^(49)^; Langsford, 2018^(50)^; Molotja, 2019^(45)^; Rendall-Mkosi *et al.*, 2013^(35)^; Sulemana *et al.*, 2013^(59)^	0	
Record-keeping of meals consumed per d/week	6	Ellis, 2012^(57)^; Fernandes *et al.*, 2017^(33)^; Hamupembe, 2016^(44)^; Moepeng, 2013^(23)^; Rendall-Mkosi *et al.*, 2013^(35)^; Sulemana *et al.*, 2013^(59)^	1	Desalegn *et al.*, 2022^(43)^
Meal diversity	5	Darko, 2014^(56)^; Khama, 2022^(49)^; Mafugu, 2021^(31)^; Moepeng, 2013^(23)^; Rendall-Mkosi *et al.*, 2013^(35)^	2	Fernandes *et al.*, 2016^(48)^; Sanousi, 2019^(53)^
Added workload for school staff	4	Banda, 2017^(42)^; Hamupembe, 2016^(44)^; Khama, 2022^(49)^; Molotja, 2019^(45)^	0	
Inconsistent meal distribution	2	Sanousi, 2019^(53)^; Sichala, 2020^(54)^		
Teachers receiving school meals when food supply is limited	2	Darko, 2014^(56)^; Rendall-Mkosi *et al.*, 2013^(35)^	0	
Absent food handlers	1	Dei, 2014^(47)^	0	
Alternative food available in/around school	1	Fernandes *et al.*, 2016^(48)^	0	
Food safety	1	Hamupembe, 2016^(44)^	0	
Encouraging students to stay all day at school	0		1	Fernandes *et al.*, 2016^(48)^
**Student stakeholders**	11		13	
Preferences	8	Banda, 2017^(42)^; Daitai *et al.*, 2018^(30)^; Ellis, 2012^(57)^; Hamupembe, 2016^(44)^; Langsford, 2018^(50)^; Mafugu, 2021^(31)^; Moepeng, 2013^(23)^; Xie & Brownell, 2020^(55)^	8	Desalegn *et al.*, 2022^(43)^, Ellis, 2012^(57)^, Khama, 2022^(49)^, Moepeng, 2013^(23)^, Molotja, 2019^(45)^, Rector *et al.*, 2021^(32)^, Sanousi,2019^(53)^, Zenebe *et al.*, 2018^(60)^
Student stigma of participation in SMP	5	Banda, 2017^(42)^; Ellis, 2012^(57)^; Hamupembe, 2016^(44)^; Khama, 2022^(49)^; Sibanda, 2012^(58)^	0	
Student perceptions of SMP (negative or positive)	1	Hamupembe, 2016^(44)^	6	Dei, 2014^(47)^; Desalegn *et al.*, 2022^(43)^; Khama, 2022^(49)^; Sanousi, 2019^(53)^; Sibanda, 2012^(58)^; Sulemana *et al.*, 2013^(59)^
Poor hygiene practices	1	Molotja, 2019^(45)^	0	
Student participation in SMP planning and organisation	0		3	Rector *et al.*, 2021^(32)^; Rendall-Mkosi *et al.*, 2013^(35)^; Xie & Brownell, 2020^(55)^
Decrease in disruptive classroom behaviour	0		1	Sanousi, 2019^(53)^
**Community involvement**	11		10	
Community engagement (parents and wider community)	6	Banda, 2017^(42)^; Hamupembe, 2016^(44)^; Khama, 2022^(49)^; Rendall-Mkosi *et al.*, 2013^(35)^; Sibanda, 2012^(58)^; Sulemana *et al.*, 2013^(59)^	1	Banda, 2017^(42)^
Community perceptions of the SMP (negative or positive)	5	Banda, 2017^(42)^; Darko, 2014^(56)^; Desalegn *et al.*, 2022^(43)^; Moepeng, 2013^(23)^; Yendaw & Dayour, 2015^(56)^	5	Banda, 2017^(42)^; Daitai *et al.*, 2018^(30)^; Moepeng, 2013^(23)^; Okae-Adjei *et al.*, 2016^(52)^; Rector *et al.*, 2021^(32)^
Shared responsibility	0		5	Molotja, 2019^(45)^; Okae-Adjei *et al.*, 2016^(52)^; Rendall-Mkosi *et al.*, 2013^(35)^; Sanousi, 2019^(53)^; Sibanda, 2012^(58)^
Employment creation	0		4	Daitai *et al.*, 2018^(30)^; Moepeng, 2013^(23)^; Rendall-Mkosi *et al.*, 2013^(35)^; Xie & Brownell, 2020^(55)^
External donors (financial and supplies)	0		3	Hamupembe, 2016^(44)^; Langsford, 2018^(50)^; Rendall-Mkosi *et al.*, 2013^(35)^
**Infrastructure support**	15		3	
Funding for SMP	9	Banda, 2017^(42)^; Hamupembe, 2016^(44)^; Langsford, 2018^(50)^; Moepeng, 2013^(23)^; Molotja, 2019^(45)^; Okae-Adjei *et al.*, 2016^(52)^; Sulemana *et al.*, 2013^(59)^; Yendaw & Dayour, 2015^(56)^	1	Rector *et al.*, 2021^(32)^
Monitoring	7	Banda, 2017^(42)^; Hamupembe, 2016^(44)^; Langsford, 2018^(50)^; Moepeng, 2013^(23)^; Molotja, 2019^(45)^; Okae-Adjei *et al.*, 2016^(52)^; Rendall-Mkosi *et al.*, 2013^(35)^	0	
Coordination	6	Hamupembe, 2016^(44)^; Khama, 2022^(49)^; Lansgford, 2018^(50)^; Moepeng, 2013^(23)^; Okae-Adjei *et al.*, 2016^(52)^; Rendall-Mkosi *et al.*, 2013^(35)^	2	Rector *et al.*, 2021^(32)^; Rendall-Mkosi *et al.*, 2013^(35)^; Xie & Brownell, 2020^(55)^
Implementation	5	Langsford, 2018^(50)^; Khama, 2022^(49)^; Rector *et al.*, 2021^(32)^; Rendall-Mkosi, 2013^(35)^; Sibanda, 2012^(58)^	2	Xie & Brownell, 2020^(55)^
Corruption	3	Langsford, 2018^(50)^; Rendall-Mkosi *et al.*, 2013^(35)^; Sanousi, 2019^(53)^	0	
SMP eligibility criteria for schools and/or students	2	Rendall-Mkosi *et al.*, 2013^(35)^; Sanousi, 2019^(53)^	1	Xie & Brownell, 2020^(55)^
Technical support and literacy among SMP stakeholders	2	Fernandes *et al.*, 2016^(48)^; Khama, 2022^(49)^	1	Rendall-Mkosi *et al.*, 2013^(35)^

Included studies varied in quality, with quantitative studies scoring Green = 1^(36)^, Amber = 4^(29,37–39)^ and Red = 2^(40,41)^, warranting careful interpretation and extrapolation of study findings. Qualitative studies mostly used acceptable/good methodological and research practices (Green = 7^(23,30,31,42–45)^, Amber = 15^(32–35,46–56)^, Red = 4^(57–60)^ (see online supplementary material, Supplemental Material 4).

### The impact of procured school meals programmes on nutritional outcomes

Overall, the results are mixed with some evidence of positive impact of publicly funded SMP on nutritional outcomes (Table 1). Subgroup analysis by gender, geography, age, socio-economic status and family composition also produced mixed results for micronutrient status, anthropometric status and dietary outcomes. Only one study reported an impact of SMP on diet and the school food environment, respectively.

#### Impact on anthropometrics

Four studies reported on anthropometric outcomes. One randomised controlled trial in Ghana^(36)^ found that SMP did not affect height-for-age Z-score (HAZ) and BMI-for-age Z-score (BAZ) in 5–15 years children participating in the SMP compared with non-participants. The authors conducted subgroup analysis on age, gender, age*gender, socio-economic status and socio-economic status*age. These analyses showed a small positive effect of the programme on HAZ among girls 5–15 years (*P* = 0·021), BAZ in boys 5–8 years (*P* = 0·028) and HAZ in all children from low-socio-economic households. In a Nigerian cohort study^(39)^, authors reported that mean weight-for-length/height Z-score in children (5–7 years) who received the SMP improved over time (–0·67 at baseline, –0·57 at 3 months, –0·41 at 6 months), while deteriorating in the control group (+0·35, –1·56, –0·17, respectively) (*P* < 0·0001). Mean weight-for-age Z-score and HAZ among beneficiaries also improved over time, while changes among non-beneficiaries were mixed (*P* < 0·0001 for both indicators). While the authors reported no statistically significant association between the SMP and wasting, they observed significant associations between the SMP and reduction in underweight (*P* = 0·001) and stunting (*P* = 0·04). Baseline data revealed major differences in nutritional status between intervention and control groups: stunting 22·0 % *v*. 44·4 %, wasting 12·0 % *v*. 6·0 % and underweight 23·2 % *v*. 2·8 %, respectively, raising concern for the comparability of included groups. Another cohort study conducted in Ethiopia^(38)^ found no significant impact of the SMP on BAZ, HAZ and anaemia in children 10–14 years. In one Kenyan^(41)^ study, children (2–10 years) who received SMP for 12 months, combined with vitamin supplementation for 3 months (when clinically required), deworming and nutrition education, were less stunted (12·0 % *v*. 22·0 %, *P* = 0·02), wasted (0 % *v*. 11·0 %, *P* = 0·02) and underweight (0 % *v*. 11·0 %, *P* = 0·06) than children of the same age who only received a deworming treatment. The proportion of children with anaemia was also lower in the intervention group compared with the control group (19·0 % *v*. 42·0 %, *P* = 0·01); however, this association is questionable as data for the intervention and control group were collected a year apart.

#### Impact on micronutrient deficiencies

Three studies reported on micronutrient deficiencies. In a randomised controlled trial in South Africa^(37)^, adding African leafy vegetables to SMP 5 days per week over 3 months reduced vitamin A deficiency in children (6–12 years) from 7·0 % (baseline) to 1·3 % (endline) in the intervention group, while no change was observed in controls (*P* = 0·015). However, this programme had no impact on iron deficiency, as the study population only had mild deficiencies at baseline, and on zinc deficiency, despite high prevalence of deficiencies in the study population. In Southern Ethiopia^(38)^, a prospective cohort study conducted over 9 months found no significant effect of the weekly menu, composed of maize, beans, cracked wheat and vegetable oil, on Hb concentration on children (10–14 years). While in Kenya^(41)^, a study conducted over 12 months reported that the prevalence of anaemia among SMP beneficiaries (children 2–10 years) was lower than non-SMP beneficiaries (*P* = 0·01) after the intervention.

#### Impact on food consumed

In a cross-sectional study in Ghana^(29)^, SMP students (12–17 years) had on average a one-unit difference in dietary diversity score at lunch compared with non-beneficiary students (7·5 *v*. 6·5 food groups, respectively, out of fourteen possible food groups, *P* < 0·001).

#### Impact on school food environment

One cross-sectional study^(40)^ reported on the impact school policies on foods sold or brought to school to limit ‘junk’ food consumption and increase fruit and vegetable intake in ninety South African schools. However, most schools had low levels of policy implementation: food regulations were used in 19 % of schools, food brought from home was checked in 13 % of schools and vegetables were featured in the school meal in 41 % of surveyed schools.

### Challenges, facilitators and authors’ recommended solutions for implementing public food procurement policies in sub-Saharan Africa schools

Facilitators were reported in twenty-three studies across the steps: *production of food* (*n* 2), *wholesale and trading* (*n* 9), *transportation and storage* (*n* 2*)*, *processing and distribution* (*n* 11), *food preparation* (*n* 9), *distribution to students* (*n* 5), *student stakeholders* (*n* 13), *community involvement* (*n* 10) and *infrastructure support* (*n* 3) (Table 3; illustrative quotes available in online supplementary material, Supplemental Material 5). Barriers were reported in twenty-six studies in each step: *production of food* (*n* 3), *wholesale and trading* (*n* 13), *transportation and storage* (*n* 13), *processing and distribution* (*n* 12), *food preparation* (*n* 17), *distribution to students* (*n* 20), *student stakeholders* (*n* 11), *community involvement* (*n* 11) and *infrastructure support* (*n* 15). Recommendations from authors of included studies were made for policy action: *production of food* (*n* 5), *wholesale and trading* (*n* 9), *transportation and storage* (*n* 5), *processing and distribution* (*n* 2), *food preparation* (*n* 15), *distribution to students* (*n* 7), *student stakeholders* (*n* 9), *community involvement* (*n* 8) and *infrastructure support* (*n* 25). Facilitators, barriers and authors’ recommended solutions (Table 4) are reported for each step of the school food system framework.

**Table 4:** Author policy-focused recommendations on implementing publicly procured school meal programmes (SMP)

Step	Studies per step in school food system	Studies
**Production of food**	6	
Support local food production	4	Darko, 2014(54); Desalegn *et al.*, 2022^(43)^; Faber *et al.*, 2013^(40)^; Sichala, 2020(55)
Government investment in agricultural sector	1	Moepeng, 2013^(23)^
Develop tools for coordinated food production for school meals programmes	1	Fernandes *et al.*, 2016^(48)^
**Wholesale and trading**	13	
Decentralise procurement (include local food)	6	Banda, 2017^(42)^; Daitai *et al.*, 2018^(30)^; Khama, 2022^(49)^; Mensah & Karriem, 2021^(34)^; Moepeng, 2013^(23)^; Sulemana *et al.*, 2013^(59)^
Support farmer collectives	2	Mensah, 2019^(51)^; Moepeng, 2013^(23)^
Develop tools to coordinate food procurement	2	Rendall-Mkosi *et al.*, 2013^(35)^; Sichala, 2020^(54)^
Transportation and storage	5	
Introduce infrastructure improvements along the supply chain	3	Desalegn *et al.*, 2022^(43)^; Faber *et al.*, 2013^(40)^; Moepeng, 2013^(23)^; Molotja, 2019^(45)^
Increase monitoring of food deliveries	3	Desalegn *et al.*, 2022^(43)^; Mafugu, 2021^(31)^; Moepeng, 2013^(23)^
**Processing and distribution**	2	
Facilitate access to school gardens	2	Faber *et al.*, 2013^(40)^; Sanousi, 2019^(53)^
**Food preparation**	15	
Implement infrastructure improvements in the school kitchen	6	Desalegn *et al.*, 2022^(38)^; Desalegn *et al.*, 2022^(43)^; Ellis, 2012^(57)^; Khama, 2022^(49)^; Molotja, 2019^(45)^; Sulemana *et al.*, 2013^(59)^
Ensure nutritious food and dietary diversity	4	Faber *et al.*, 2013^(40)^; Hamupembe, 2016^(44)^; Khama, 2022^(49)^; Van der Hoeven *et al.*, 2015^(37)^
Train food handlers	4	Banda, 2017^(42)^; Desalegn *et al.*, 2022^(38)^; Khama, 2022^(49)^; Mafugu, 2021^(31)^
Ensure meal quantity and quality requirements are met	3	Moepeng, 2013^(25)^; Yendaw & Dayour, 2015^(56)^
Pay food handlers	2	Khama, 2022^(49)^; Rendall-Mkosi *et al.*, 2013^(35)^
Develop tools for coordination of meal preparation	2	Hamupembe, 2016^(44)^; Mafugu, 2021^(31)^
Find regular external donors/funders	1	Hamupembe, 2016^(44)^
**Distribution to students**	8	
Introduce infrastructure improvements for food service and student dining spaces	4	Desalegn *et al.*, 2022^(43)^; Ellis, 2012^(57)^; Khama, 2022^(49)^; Molotja, 2019^(45)^
Monitor handwashing among students before food service	3	Ellis, 2012^(57)^; Hamupembe, 2016^(44)^; Khama, 2022^(49)^
Allot time in school day for school meal	2	Banda, 2017^(42)^; Sulemana *et al.*, 2013^(59)^
Ensure healthy food environment in school	1	Fernandes *et al.*, 2017^(33)^
**Student stakeholders**	9	
Implement nutrition education programme	5	Daitai *et al.*, 2018^(30)^; Desalegn *et al.*, 2022^(38)^; Faber *et al.*, 2013^(40)^; Rendall-Mkosi *et al.*, 2013^(35)^
Teach school gardening as specific topic	4	Khama, 2022^(49)^; Rendall-Mkosi *et al.*, 2013^(35)^; Sibanda, 2012^(58)^; Xie & Brownell, 2020^(55)^
Teach food production as specific topic	3	Faber *et al.*, 2013^(40)^; Khama, 2022^(49)^; Molotja, 2019^(45)^
Assess nutritional status of students	1	Van der Hoeven *et al.*, 2015^(37)^
**Community involvement**	8	
Encourage community participation	6	Ellis, 2012^(57)^; Hamupembe, 2016^(44)^; Moepeng, 2013^(25)^; Molotja, 2019^(45)^; Okae-Adjei *et al.*, 2016^(52)^; Yendaw & Dayour, 2015^(56)^
Introduce a school meal programme awareness campaign	3	Sichala, 2020^(54)^; Sulemana *et al.*, 2013^(59)^; Yendaw & Dayour, 2015^(56)^
**Infrastructure support**	25	
Provide funding and resources (infrastructure improvements, increase programme funding and resources (including training), pay and regulate food suppliers and food handlers, review external partnerships, programme roll-out)	16	Darko, 2014^(56)^; Desalegn *et al.*, 2022^(43)^; Ellis, 2012^(57)^; Hamupembe, 2016^(44)^; Khama, 2022^(49)^; Mafugu, 2021^(31)^; Mensah, 2019^(51)^; Moepeng, 2013^(23)^; Molotja, 2019^(45)^; Oyela *et al.*, 2023^(39)^; Rendall-Mkosi *et al.*, 2013^(35)^; Sanousi, 2019^(53)^; Sibanda, 2012^(58)^; Sulemana *et al.*, 2013^(59)^; Yendaw & Dayour, 2015^(56)^; Zenebe *et al.*, 2018^(60)^
Implement monitoring and evaluation (surveillance, evaluation, research, reporting)	11	Banda, 2017^(42)^; Daitai *et al.*, 2018^(30)^; Darko, 2014^(56)^; Desalegn *et al.*, 2022^(38)^; Hamupembe, 2016^(44)^; Khama, 2022^(49)^; Molotja, 2019^(45)^; Oyela *et al.*, 2023^(39)^; Rendall-Mkosi *et al.*, 2013^(35)^; Sanousi, 2019^(53)^; Sibanda, 2012^(58)^
Strengthen leadership (awareness campaign, creation and/or re-examination of SMP policy, re-evaluation of programme scope and reach, political commitment)	9	Abizari *et al.*, 2021^(29)^; Ellis, 2012^(57)^; Faber *et al.*, 2013^(40)^; Fernandes *et al.*, 2017^(33)^; Hamupembe, 2016^(44)^; Khama, 2022^(49)^; Moepeng, 2013^(23)^; Rendall-Mkosi *et al.*, 2013^(35)^; Sibanda, 2012^(58)^; Sulemana *et al.*, 2013^(59)^; Xie & Brownell, 2020^(55)^
Introduce platforms for interaction (coordination, create and/or follow national nutrition guidelines, tools to organise)	5	Daitai *et al.*, 2018^(30)^; Fernandes *et al.*, 2017^(33)^; Moepeng, 2013^(23)^; Molotja, 2019^(45)^; Rector *et al.*, 2021^(32)^
Strengthen governance (include stakeholders at all programme stages, regulate school food environment)	4	Faber *et al.*, 2013^(40)^; Molotja, 2019^(45)^; Okae-Adjei *et al.*, 2016^(52)^
Introduce health in all policies (health and nutrition included in the agenda of all ministries)	4	Moepeng, 2013^(23)^; Molotja, 2019^(45)^; Rector *et al.*, 2021^(32)^; Sichala, 2020^(54)^

### Production of food

Switching SMP from centrally sourced to domestically grown crops was a challenge for countries, like Namibia, where imported food makes up the majority of available food^(57)^. Promoting local procurement in Botswana sought to create more reliable market access for farmers^(34)^ increasing economic activity and sometimes crop diversification^(23)^. However, government budgets for local production were low, and only individual farmers (not farmer groups) could apply for contracts, making it difficult to supply sufficient quantities of crops that met set quality standards^(23)^.

*Authors’ recommended solutions* include creating links with farmers to promote more local and sustainable procurement approaches^(23,40,43,46,48,54)^. Examples include supporting local agriculture production of micronutrient rich vegetables and incorporating them in meals served^(43)^ and including tools, such as ‘The School Meals Planner Package’ in Ghana, to include local produce in weekly menus^(48)^.

### Wholesale and trading

In terms of wholesale and trading, no consensus on best type of procurement model was reached. Centralised procurement was challenging when the food supply was disrupted, as it impacted the entire programme^(42)^. However, moving away from centralised to decentralised procurement was also cited as a challenge^(49,50)^. For example, including or increasing the percentage of locally procured food for SMP was difficult, particularly due to the seasonality, quality or scale of local production, especially in non-agricultural regions^(23,30,35,45,57)^. Alternatively, non-local procurement was problematic as it failed to support the local economy, with school menus composed of non-traditional and international foods such as tinned fish and soya.

Depending on the context, some sources reported that centralised procurement was a SMP facilitator, as the directives of the overall programme came from one source, facilitating programme management, purchasing, implementation and reporting^(23)^. Competitive market prices, offered to those buying large quantities, often reduced financial burden of schools, thus ensuring equal access to food, even among remote schools^(23,35,45)^. Other sources reported the advantages of decentralised procurement models, which allowed schools to have more flexible procurement criteria. Decentralised models allowed SMP to set budgets in advance, which helped reduce corruption^(32,34,35,50)^. Meanwhile, some centralised models included local food, allowing schools to manage SMP independently, encouraging local purchasing and reducing costs for food-related transportation^(23,34,35,43,51)^. Freshness of local food was positively associated with locally grown crops, with some studies noting that students preferred these foods^(23,34,35,43,51)^. Additional challenges included delayed contracts, supplier payments and changing class sizes and enrolments throughout the year, making food orders complex^(23,49)^. On the other hand, establishing contracts with food providers helped ensure on-time deliveries, food quality and financial transparency, such as a public record of purchasing to facilitate SMP’ implementation^(31,45)^.

*Authors’ recommended solutions* included changes to current procurement models, such as a shift from centralised to decentralised modalities to promote, or require, locally produced food in SMP^(23,30,34,42,49,59)^. Creating farmer cooperatives and grain banks was also suggested to support smallholder/local farmers^(23,51)^.

### Transportation and storage

Irregular, inadequate and/or late food deliveries represented real challenges that disrupt SMP and make nutritional gains difficult to achieve and record^(23)^. Irregular deliveries were linked with poor food procurement processes and seasonality^(30,34,42,50,58)^. However, large food deliveries and improved infrastructure could safeguard against delays^(44,54)^. The poor quality of food delivered was concerning, with four studies^(23,43–45)^ detailing that food was often spoiled upon delivery, highlighting logistic challenges of food storage during transportation in local and national supply chains^(23,49)^.

*Authors’ recommended solutions* included developing applications to communicate and track food delivers, monitor the quality of food deliveries, monitor store room inventories and improve storage facilities^(23,31,40,43,45)^.

### Processing and distribution

Facilitators for processing and distribution included good programme coordination, motivated and dedicated school staff, adequate food storage and contracting food suppliers trained in food safety and hygiene^(23,30,32,34,45,47,49,53)^. Record-keeping also helped to ensure adequate food supply by facilitating monitoring efforts and increasing the frequency of reports. Inadequate on-site school storage was cited as a challenge in twelve studies^(30,31,35,42–45,47,49,55,57,60)^. While some schools described on-site storage facilities, these were often poorly adapted for hygienic food storage. Poor ventilation, storing food on the floor and using classrooms as makeshift storage spaces were listed as unsafe food storage practices, creating opportunities for food spoilage, theft and burglary. In addition, school gardens were also viewed as facilitators, contingent on land availability and production rates (i.e. enough fruit and vegetables to complement SMP)^(32,35,45)^, but as a burden when poorly maintained and not included in educational activities^(30,32,42,53,57)^.

*Authors’ recommended solutions* detailed developing or improving existing school gardens to supplement fruit and vegetable procurement in SMP and budgeting for upkeep^(40,53)^.

### Food preparation

In many countries, mothers of children attending the school became the school cook. These mothers were often viewed as unpaid volunteers. However, not paying wages resulted in delays in meal preparation and even programme suspension. Among paid food handlers, delayed payments from the government posed a challenge for purchasing food, in turn causing delayed payments to suppliers^(51)^. Training on safe food handling was not uniform or compulsory and thus, an additional challenge. Concerns among parents and students were raised about the lack of training on food safety among food handlers in nine studies^(31,32,42–44,49,50,52,55,60)^. Meal preparation often represented a large workload, demanding time and energy to cook. Some students in Zambia were tasked with meal preparation when food handlers were absent, taking them away for their studies^(42)^. Additional food preparation challenges reported were a lack of school meal guidelines and infrequent record-keeping of meals prepared and ingredients used. Food preparation was facilitated by reliable infrastructure, such as well-designated and clean spaces to store and cook food, reliable and paid food handlers, food safety training, medical certifications, food measurement and school guidelines, such as weekly menus^(48)^.

*Authors’ recommended solutions* comprised hiring trained and paid food handlers for food preparation to ensure safe and uninterrupted meal service^(29,33,36,41,51,59)^. Several studies also recommended infrastructure improvements, specifically for kitchen equipment and designated cooking spaces^(38,43,45,49,57,59)^; one study suggested establishing a partnerships with local funders^(44)^. School feeding manuals and platforms to input attendance records and ways to track student participation during meals were also recommended^(31,44)^. Improvement of meal quantity and nutritional quality, including nutritionally adequate and diverse food groups, such as fruit and vegetables, was widely advised^(23,37,40,44,49,56)^.

### Distribution to students

Overall food distribution challenges included irregular meal services, meals served in unhygienic and unsafe spaces and a lack of programme monitoring and record-keeping of food distributed and consumed. Designated school canteen spaces were rare, resulting in students eating outdoors, often on the ground. A major challenge during meal distribution was poor nutritional quality, diversity and quantity, with portions getting smaller towards the end of lunchtime^(23,30,35,43,45–47,49,52–54,57,59)^. Establishing a dedicated school breakfast or lunch period was also a challenge. In several studies, school staff complained that SMP reduced teaching time or added to their overall workload, while others reported that lunch periods were too short for students to eat a full meal. This challenge was also linked to students not having proper cutlery and bowls, with some waiting for their friends to finish before borrowing them for use.

Conversely, food distribution was facilitated when daily attendance and daily meal participation were recorded^(43)^, utensils/crockery were provided^(57)^ and leftover/take-home rations were given to vulnerable students^(53)^. Distributing food to students increased daily food intake and motivated students to stay at school for the entire day, avoiding travelling home for lunch, especially when meals were varied throughout the week^(33,53)^. Universal eligibility among children was reported to reduce the stigma associated with eating free meals^(55)^.

*Authors’ recommended solutions* to improve food distribution included enhancing serving and dining facilities and providing bowls and cutlery for all students^(43,45,49,57)^. Monitoring handwashing before meals and allocating a designated mealtime were also recommended^(44,49,57)^.

### Student stakeholders

Students’ dislike of some school meals (e.g. beans, soya-based), resulting in reduced participation, was cited in eight studies^(23,30,31,42,44,50,55,57)^. Poor hygienic practices, such as not washing hands before eating, were also a barrier to programme implementation^(45)^. Considering student preferences when creating school menus and offering membership to school gardening clubs encouraged student participation, allowing them to become active stakeholders and facilitating implementation^(32,55)^. Inversely, eight studies reported that students liked having a school meal^(23,32,43,45,49,53,57,60)^, and increased participation was observed when students perceived links with positive educational, health or nutritional outcomes^(45)^.

*Authors’ recommended solutions* included revising the school curriculum to include nutrition education^(59)^, with specific content on hygiene practices^(35)^, agriculture and food production^(40,45,49)^ and school gardens^(35,49,55,58)^. One study also recommended regularly assessing students’ nutritional status^(37)^.

### Community involvement

Communities had negative perceptions of SMP when parents considered meals to be of low nutritional quality (e.g. few fruit and vegetables served) and quantity^(23)^. However, among communities where nutritional and educational gains were observed, particularly in reducing short-term hunger, SMP were more successful. The role of SMP as a social security net to support household food security was also discussed^(52)^. Little to no information sharing led to low levels of parental involvement and unengaged community members^(58)^. Hiring community members, often mothers, to work in SMP provided local employment opportunities and further promoted household food security^(55)^. In addition, working with external funders in the community helped improve SMP infrastructure, by constructing permanent kitchens or purchasing cutlery^(35)^.

*Authors’ recommended solutions* consist of introducing national and local awareness campaigns on SMP objectives to increase community support and engagement^(54,56,59)^.

### Infrastructure support

Lack of policy and legislation for funding, coordination, implementation, monitoring, corruption, eligibility and technology were notable challenges. Inadequate programme funding was cited as a challenge by nine studies^(23,42,44,45,50,52,56,59)^. At the national, regional and school levels, SMP coordination was cited as a challenge, especially when no dedicated coordinating agency or branch of government was charged with programme oversight. Poor programme coordination led to gaps in implementation, leaving room for incorrect food orders, late deliveries, corruption and placing more responsibility on school staff^(23,32,35,44,49,50,52,58,58)^. Large distances between schools and damaged and/or bottlenecked roads created additional logistical challenges for SMP staff. Furthermore, in programmes with policies and guidelines, monitoring and evaluation efforts revealed low levels of implementation^(23,35,42,44,45,50,52)^. Corruption was also cited as a challenge, mainly attributed to large SMP budgets. In South Africa, where eligibility was based on the *quintile system*, classifying schools by proxy of children’s socio-economic status was a barrier to ensuring that all children in need qualified for school meals^(35,53)^.

Infrastructure support facilitated implementation when financial and technical support from the government, such as capacity building workshops, food handler training and programme monitoring, was provided. Additionally, using models of best practice, successful programming and guidelines, like national nutrition requirements, facilitated procurement^(32,35,55)^. Additionally, tools to measure standard quantities, records of attendance and software applications, like the School Meals Planner in Ghana, helped food handlers design menus to meet nutrition requirements and procurement officers determine the correct quantities to purchase within allocated budgets^(48)^.

*Authors’ recommended solutions* encompassed all aspects of infrastructure support. Overall political will and leadership commitment to SMP were key recommendations^(35,59)^. High-level political champions of SMP, such as the First Lady of Ethiopia, also encouraged success^(55)^. Introducing national awareness campaigns on SMP objectives and the importance among programme beneficiaries and stakeholders was also proposed^(35,44,57–59)^. Four studies recommended developing a national policy to guide their SMP^(23,44,49,57)^, while two additional studies recommended implementing a unified procurement framework and/or guiding document to help tailor policy design to focus on children/adolescents^(33,55)^.

In terms of recommendations to strengthen governance, one South African study suggested requiring national regulations for food sold in/around schools^(40)^. Others recommended revising educational curricula to include SMP and local food production^(23,32,45,54)^ and working with qualified nutritionists, health professionals and/or professional chefs, to create nutrition-based recommendations and SMP guidelines^(45,52)^. Recommendations for improved monitoring and evaluation included establishing public records of transactions to reduce corruption^(30,35,38,39,42,44,45,49,57–59)^, as well as suggestions to streamline monitoring meal quantity and quality^(46,53)^. These recommendations go alongside the need to improve platforms for interaction and ways to make policies and actions coherent between different levels of government^(30,45)^; perhaps developing national nutrition guidelines and food composition tables^(32,48)^ or using information management systems^(23,45)^ could be used to achieve these goals.

Finally, recommendations were made to ensure that funding and resources were sufficient to employ and train more personnel to meet programme needs^(23,35,43,45,49,57,58)^. Funding infrastructure improvements in schools, including sanitation and agricultural inputs^(35,44,59)^ and on-time payments to food suppliers and food handlers, could allow SMP to run smoothly^(31,44,46,49,51,59)^. Increased technological support for SMP officers and school staff was proposed to ensure the proper use of online ordering systems and programme coordination tools^(46,52)^. Additionally, revising SMP eligibility criteria for funding to ensure the long-term viability across varied contexts and expanding programme reach was recommended^(39,43,46,53,58,60)^. Creating partnerships with NGO and other stakeholders to fundraise or increase support was also suggested^(35,51,59)^.

## Discussion

While studies linking SMP and nutritional outcomes were found in nine SSA countries, the extent of evidence was limited. Evidence from seven studies on the impact of publicly funded SMP and nutrition outcomes was mixed, explained in part, by inadequate research designs used to evaluate impact. Future experimental research studies should not only consider improving research design and increasing the intervention period but also fully consider ethical implications^(61)^. Malnourished school children represent one of the world’s most vulnerable populations and studies need to be rigorously designed to address objectives and ensure that children’s engagement is best valued. Evidence from twenty-six qualitative studies concluded that developing or revising publicly procured SMP to include healthy (nutritious and safe) food at all levels of the school food system has potential, particularly when included in overall programming and at each step of implementation.

This review chose to use the term ‘school meals’ to refer to all school-based food provision programmes, as opposed to ‘school feeding’, because the term ‘feeding’ implies a passive action. As the results highlight, students are not passive programme beneficiaries but active participants. Several studies suggested that neglecting student preferences and opinions limited programme success^(32,35,55)^, especially when older children and adolescents are consulted, which is a key consideration for future programming creation and modification.

### Policy implications

There is a global shift towards more decentralised procurement. However, evidence from this review suggests that no single procurement method works best in SSA. In some settings, centralised models allow SMP to thrive, as all logistics are organised at the highest level and all schools receive the same materials and food supply. Centralised procurement can present opportunities to include healthy food, such as fruit and vegetables, in SMP with few changes to national guidelines. The centralised model in Botswana began working with Botswanan farmers to include local foods, like melon, in school meals, demonstrating that locally sourced foods can also be included in this type of procurement model^(23)^.

Alternatively, decentralised models, with flexible procurement requirements for smallholder farmers may be preferable in countries that seek to focus on building smaller scale and/or sustainable community models. Notwithstanding, decentralisation can pose a risk to SMP, as this procurement model shifts food procurement responsibilities to lower administrative levels and often to individual schools, which may overwhelm staff. Trade-offs are important to consider, especially if training and resources are not provided during programme transitions^(51,55,61)^. South Africa, for example, has a dedicated SMP unit within in the Department of Education, but unreliable funding and limited staff hinder programme implementation^(1,35)^. Further research to explore the underlying mechanisms that determine which procurement model is best suited for each country is of merit and should be considered alongside each country’s objectives.

Regardless of the selected procurement model, governments with SMP should introduce legislation to structure each programme and commit to a dedicated line for school meals in the national budget. Programme buy-in from the Ministry of Education is key to SMP’ success^(61)^ alongside staff engagement and motivation. The Government of Ghana, for example, declared its commitment to create a national food procurement policy focused on including healthy food service in schools. Therefore, the Ghanaian government developed food-based dietary guidelines in 2022 and is in the process of developing a nutrient profiling model to facilitate the implementation in all food-based policies^(62)^. Countries that are in the process of selecting or restructuring existing procurement model for SMP can use an existing set of tools compiled by the African Union Development Agency (AUDA-NEPAD) and the WHO as well as case studies by the World Food Programme (WFP) and the School Meals Coalition to ensure that the selected model works well for all SMP stakeholders^(11,13,63,64)^.

In addition to revising allocated budgets and nutritional content of SMP, investment to improve the infrastructure surrounding SMP is needed to support farmers, wholesalers, school cooks and programme staff. Without improved roads, food deliveries may continue to arrive with delays, putting pressure on school staff and taking away learning time^(44,54)^. Unreliable road infrastructure could also jeopardise programme monitoring and evaluation efforts as staff cannot easily travel between schools. Investments in national electricity grids and provision of clean water are key priorities to ensure the timely delivery of safe school meals. Additionally, connections to electricity could increase the use of refrigeration of perishable food items, thus improving storage conditions across several steps of the food system. For example, storage facilities, such as school kitchens, should be equipped with a refrigerator or freezer to increase the inclusion of vegetables and animal-sourced foods in the meals while simultaneously reducing food spoilage^(23,49)^ and waste.

### Strengths and limitations of the review

This is the first review of publicly procured food in SSA, and it includes an abundance of rich qualitative data on the subject. Limitations of this review are attributable to the heterogeneity of included studies (in terms of outcomes, targets, methodology and quality), which made quantitative evidence synthesis difficult and removed the possibility to conduct a meta-analysis to draw firm conclusions. Additional nationally funded SMP in SSA are known^(8)^, but no studies from these countries were identified. Furthermore, no publications in French were identified. However, many Francophone SMP were recently expanded, and research or programme evaluation may be forthcoming.

## Conclusion

While several studies recommend more rigorous research to measure nutritional outcomes, we recommend improving the overall structure of SMP and ensuring effective programme implementation before undertaking large-scale trials. Before the quality of evidence collected can improve, programme coordination and monitoring need to be implemented and overseen. SMP stakeholders including different governmental ministries (i.e. agriculture, education and health) need to collaboratively and synergically provide programme support. For example, this review suggests that while improvements are needed across the school food system, strengthening *infrastructure support* and *food preparation*, followed by *student engagement* and *wholesale and trading*, should be prioritised. This can be done by introducing or updating the national SMP policy to include criteria for nutritious school meals. Increased commitment to programme monitoring and evaluation, such as improved record-keeping for food delivered, prepared and consumed, is also recommended. As nutritional quality and quantity of school meals were also highlighted as a challenge, using dietary guidelines can be used to promote the inclusion of nutritionally adequate and diverse food groups, such as fruit and vegetables, in SMP across SSA. While the creation and use of electronic tools to share data is recommended to facilitate this process, training and technical support will also be required and should be budgeted for accordingly. Cost estimates, dedicated annual funding and governments’ renewed commitment are all necessary to ensure that the nutritional quality and safety of food served in SMP are guaranteed before expanding coverage and scaling up.

## Supporting information

Liguori et al. supplementary material 1 Liguori et al. supplementary material

Liguori et al. supplementary material 2 Liguori et al. supplementary material

Liguori et al. supplementary material 3 Liguori et al. supplementary material

Liguori et al. supplementary material 4 Liguori et al. supplementary material

Liguori et al. supplementary material 5 Liguori et al. supplementary material

## Supplementary material

For supplementary material accompanying this paper, visit https://doi.org/10.1017/S1368980024001939.
