# Supplementary material for: How do publicly procured school meals programmes in sub-Saharan Africa improve nutritional outcomes for children and adolescents: a mixed-methods systematic review
Source: Public Health Nutr. 2024 Oct 18;27(1):e213. doi: 10.1017/S1368980024001939 (PMC11604325; doi:10.1017/S1368980024001939)
Supplement: Liguori et al. supplementary material 1 — Liguori et al. supplementary material [file S1368980024001939sup001.docx]

**Supplementary File 1.** **Eligibility criteria used in the systematic review**

|  | Inclusion Criteria | Exclusion Criteria |
| --- | --- | --- |
| Population | Children and adolescents (5-19) in primary or secondary schools | Infants and young children under 5; out of school children and adolescents (5-19) |
| Intervention | Public procurement of food in a school (school meal provision: breakfast, lunch, snack, take home ration) | Private funded procurement (e.g., corporate initiatives), interventions at home or community level only, micro-nutrient supplementation only programmes, nutrition education only programmes |
| Comparison (Context) | Sub-Saharan Africa | North Africa and countries in any other world region |
| Outcome | Food environment or physical nutritional outcomes, including body mass index (BMI), underweight, overweight/obesity and micronutrient deficiencies, minimum dietary diversity (MDD-W), Global Diet Quality Score, consumption of micronutrient-rich foods and challenges and facilitators to implementation of public procurement policies and school meal programmes | Education only outcomes; non-nutrition/health related outcomes |
| Study type | Randomised & non- randomised controlled trials, quasi randomised trials, prospective cohort studies with repeated cross-sectional design, qualitative studies, mixed method studies | Non-human studies |
| Publication type | Peer-reviewed journal articles; grey literature including: master thesis, doctoral thesis, report, working papers | Book, conference abstract, meta-analysis, methods paper, review, website |
| Publication | 2011-2023 | Before 2011 |
| Language | English and French | Languages other than English and French |
