# Supplementary material for: How do publicly procured school meals programmes in sub-Saharan Africa improve nutritional outcomes for children and adolescents: a mixed-methods systematic review
Source: Public Health Nutr. 2024 Oct 18;27(1):e213. doi: 10.1017/S1368980024001939 (PMC11604325; doi:10.1017/S1368980024001939)
Supplement: Liguori et al. supplementary material 2 — Liguori et al. supplementary material [file S1368980024001939sup002.docx]

**Supplementary File 3: List of excluded articles at the data extraction stage and reasons**

| **Author** | **Year of publication** | **Title** | **Country** | **Reason for exclusion** |
| --- | --- | --- | --- | --- |
| Appleby | 2019 | Integrated delivery of school health interventions through the school platform: Investing for the future | Ethiopia | No nutrition outcome |
| Abizari et al. | 2014 | School feeding contributes to micronutrient adequacy of Ghanaian schoolchildren | Ghana | No nutrition outcome, cross-sectional only measuring prevalence of malnutrition at one point in time |
| Bekker et al. | 2017 | The provision of healthy food in a school tuck shop: does it influence primary-school students’ perceptions, attitudes and behaviours towards healthy eating? | South Africa | Not public procurement, focus on school tuck shops |
| De Villiers et al. | 2012 | “HealthKick”: Formative assessment of the health environment in low-resource primary schools in the Western Cape Province of South Africa | South Africa | Not public procurement, HealthKick Project |
| Devereux et al. | 2018 | School Feeding in South Africa: What we know, what we don’t know, what we need to know, what we need to do | South Africa | Not original research, focus on public-private partnerships |
| Gelli et al. | 2016 | Evaluation of alternative school feeding models on nutrition, education, agriculture and other social outcomes in Ghana: rationale, randomised design and baseline data | Ghana | No nutrition outcome, baseline data and set up of a RCT, endline data was included in the review in Gelli et al., 2019 |
| Gewa et al. | 2011 | A school based supplementary food programme in rural Kenya did not reduce children's intake at home | Kenya | Not public procurement, not part of a public meal programme, study conducted in 1998-2000 |
| Graham et. Al | 2015 | Evaluation study of the national school nutrition programme and the Tiger Brands Foundation in-school breakfast feeding programme in the Lady Frere and Qumbu districts of the Eastern Cape | South Africa | Not public procurement, this programme is privately funded by a corporate initiative, Tiger Brands Foundation Project |
| Makanu et al. | 2022 | Determinants of dietary patterns in school going adolescents in Urban Zambia | Zambia | Not public procurement, cross-sectional prevalence of malnutrition |
| Masset and Gelli | 2013 | Improving community development by linking agriculture, nutrition and education: design of a randomised trial of “home-grown” school feeding in Mali | Mali | No nutrition outcome, baseline data and set up of a RCT, the trial was not finished due to civil conflict in Mali |
| Marraccini et al. | 2012 | A Qualitative Evaluation of Exposure to and Perceptions of the Woolworths Healthy Tuck Shop Guide in Cape Town, South Africa | South Africa | Not public procurement, focus on school tuck shops |
| Muhirwe et al. | 2021 | Experiences from implementation of a school based nutrition programme in Wakiso District, Central Uganda | Uganda | Not public procurement, though supported by the government in some aspects, the school meal was based on parent-led participation |
| Steyn et al. | 2015 | Did HealthKick, a randomised controlled trial primary school nutrition intervention improve dietary quality of children in low-income settings in South Africa? | South Africa | Not public procurement. HealthKick Programme |
| Wangart et al. | 2020 | Changing Perception through a Participatory Approach by Involving Adolescent School Children in Evaluating Smart Food Dishes in School Feeding Programs – Real-Time Experience from Central and Northern Tanzania | Tanzania | Not public procurement |
| Zwane | 2014 | School feeding programmes as a mechanism to improve market access for smallholder farmers in rural areas of South Africa | South Africa | No nutrition outcome, focus on smallholder farmers |
