# Supplementary material for: How do publicly procured school meals programmes in sub-Saharan Africa improve nutritional outcomes for children and adolescents: a mixed-methods systematic review
Source: Public Health Nutr. 2024 Oct 18;27(1):e213. doi: 10.1017/S1368980024001939 (PMC11604325; doi:10.1017/S1368980024001939)
Supplement: Liguori et al. supplementary material 3 — Liguori et al. supplementary material [file S1368980024001939sup003.docx]

**Supplementary File 4: MMAT Quality Appraisal**

| **Author** | **Title** | **Year of publication** | **Type of appraisal** | **Final colour agreed upon by both appraisers** | **Reasons for the colour given** |
| --- | --- | --- | --- | --- | --- |
| Banda, 2017 | An evaluation of the implementation of home-grown school feeding programme in selected primary schools in Nimbi District, Zambia | 2017 | 1. Qualitative | Green | Well-presented study. Could provide more details for the methods section on analysis. |
| Daitai et al., 2017 | The effects of the school feeding programmes in addressing food insecurity in Mutale Municipality of Vhembe District, Limpopo Province. | 2017 | 1. Qualitative | Green | Thorough case study, transparent on methods (sampling, participants etc); includes linkages between theory and findings; interviews with diverse stakeholders; covers broad topics around implementation of school meals. Could provide more details on qualitative analysis. |
| Darko, 2014 | The school leadership views on the impact of the national school feeding programme in Ghana: the case study of Adumanu M/A and Ayaasi D/A primary schools | 2014 | 1. Qualitative | Amber | Analysis seems well conducted, but there is a small sample size and views of only the school leadership are presented in the results. |
| Dei, 2014 | An evaluation of the school feeding programme: A case study of Magog primary school, University of South Africa | 2014 | 1. Qualitative | Amber | The case-study provided an in-depth view of the functioning of the school meal programme at one school. However, the methods section on data collection and analysis could be clearer to show what was actually done. Some variables/information is missing in their extraction sheet that could have been helpful to have. |
| Desalegn et al., 2022 | Successes and challenges of the home-grown school feeding program in Sidama Region, Southern Ethiopia: A qualitative study | 2022 | 1. Qualitative | Green | Well-presented and explained study. Could be strengthened with the inclusion of some of the national partners in the SFPs, such as the Ministry of Education, and the international partners, including FAO and WFP. |
| Ellis, 2012 | The Namibian school feeding Programme: A case study | 2012 | 1. Qualitative | Red | Primary findings are not presented. The sample characteristics of each focus groups are not included. |
| Fernandes et al., 2017 | A free lunch or a walk back home? The school food environment and dietary behaviours among children and adolescents in Ghana | 2017 | 1. Qualitative | Amber | The study conducted surveys in 10 regions, but only seems to have reported on and used quotes from two of the regions to explain the findings all 10 regions. |
| Fernandes et al., 2016 | Enhancing linkages between healthy diets, local agriculture, and sustainable food systems: The school meals planner package in Ghana | 2016 | 1. Qualitative | Amber | The methodology section on the different components of the tool is very clear, but the way they interviewed stakeholders to find out how the tool is used/received is missing. Limitations of qualitative data analysis not clearly presented. Who participated in the focus groups is not reported clearly. It is stated that 29 observations were done, but were these observations made once at each school or across the schools. The presence of the observer might have encouraged the use of the tool and is not reported on. |
| Hamupembe, 2016 | Investigating the administration of the school feeding programme: A case study of two primary schools in Windhoek, Namibia | 2016 | 1. Qualitative | Green | Well conducted thesis. Supporting evidence is well presented. |
| Khama, 2022 | Experiences of the implementers of the school feeding programme in two schools in the Zambezi region, Namibia | 2022 | 1. Qualitative | Amber | Detailed case-study. Limited number of interviews and the same quotes were used to explain different themes/make the main points of the paper. |
| Langsford, 2018 | Enough on our plate? The national school nutrition programme in two schools in Katlehong, South Africa | 2012 | 1. Qualitative | Amber | The analysis could be stronger and more clearly described. Use of direct quotes instead of only summaries would also strengthen this paper. Otherwise, this is a detailed case study with onsite observations. |
| Mafugu, 2021 | Challenges encountered in a South African school nutrition programme | 2021 | Qualitative | Green | Strong methodology and results are well presented with supporting citations from study participants. |
| Menash & Karriem, 2021 | Harnessing public food procurement for sustainable rural Livelihoods in South Africa through the national school nutrition programme: A qualitative assessment of contributions and challenges | 2021 | 1. Qualitative | Amber | Primary data included with original quotes. The methods are reasonably well described, but sampling is confusing: how many interviews were conducted/with whom. Lacking information on the way that the data was analysed. There are direct quotes used, but their presentation only seems to partially support the narrative presented. |
| Mensah, 2019 | Incentivising smallholder farmer livelihoods and constructing food security through home-grown school feeding: evidence from Northern Ghana | 2019 | 1. Qualitative | Amber | The methods and results sections are poorly presented. The data on caterers seem to be more compressive than other groups, for example age and gender are reported. Few quotes. Quotes representing all participant groups are missing. |
| Moepeng, 2016 | Botswana national primary school feeding programme: A case study. | 2016 | 1. Qualitative | Green | Comprehensive report with strong methodology. Evidence could be strengthened by providing more quotes and more information on observations. |
| Molotja, 2019 | Towards a framework for enhancing school feeding programmes for rural development in Blouberg Local Municipality, South Africa | 2019 | 1. Qualitative | Green | Well conducted study. |
| Okae-Adjei et al., 2016 | Ghana’s school feeding programme in perspective: A case study of the Akuapem North Municipality in the Eastern Region | 2016 | 1. Qualitative | Amber | Evidence to support data is limited, used mixed methods to try to better understand stakeholders’ views on the changes in students since school meals were introduced. Methods and analysis could be better explained and results strengthened with the addition of participant quotes. |
| Rector et al., 2021 | School-Based nutrition programs for adolescents in Dodoma, Tanzania: A situation analysis | 2021 | 1. Qualitative | Amber | Insufficient detail given on source of findings as they are presented as aggregated findings, mainly, without individual quotations; sample not accurately described. |
| Rendall-Mkosi, 2013 | Case study of the national school nutrition programme in South Africa; University of Pretoria: | 2013 | 1. Qualitative | Amber | Thorough case study, but missing some details. For example, how the study was actually conducted, with how many participants and the type of analysis used to reach their conclusions are not explained. |
| Sanousi, 2019 | The expected effects of the national school nutrition programme: Evidence from a case study in Cape Town, Western Cape | 2019 | 1. Qualitative | Amber | The study aim is clear, methods are well presented and barriers of the NSNP and solutions reported. However, several limitations were recorded or reported. Analysis is not described. |
| Sibanda, 2012 | An analysis of the implementation of the school supplementary feeding programme in Windhoek, Namibia | 2012 | 1. Qualitative | Red | The overall text could be much clearer, with the methods clearly presented. Based on one focus group with eight participants. |
| Sichala, 2020 | Food for education programme and prospects for multi-sectoral gains: experiences from Kazungula and Sinazongwe districts of Southern Zambia | 2020 | 1. Qualitative | Amber | Methods could be strengthened or reported more clearly. Results provide good insight and are well presented. |
| Sulemana et al., 2013 | The challenges and prospects of the school feeding programme in Northern Ghana | 2013 | 1. Qualitative | Red | There is a lack of transparency of methods. Unclear if primary data is presented, seems to be a summary of all interviews and focus groups. A table of study participants is not included (do not know how many from each category participated). |
| Xie & Brownell, 2020 | Nutritious food procurement in cities in low and middle income countries case studies on Addis Ababa students feeding agency and Pune Sassoon General Hospital meal programme | 2020 | 1. Qualitative | Amber | Key messages are easy to find and clear, but we don't know the sources/participants. Limited number of interviews and limited number of types of respondents |
| Yendaw & Dayour, 2015 | Effect of the national school feeding programme on pupils‟ enrolment, attendance and retention: A case study of Nyoglo of the Savelugu-Nantong Municipality, Ghana. | 2015 | 1. Qualitative | Amber | Detailed results presented, methods section and type of analysis used could be further clarified. |
| Zenebe et al., 2018 | School feeding program has resulted in improved dietary diversity, nutritional status and class attendance of school children | 2018 | 1. Qualitative | Red | Qualitative findings not sufficiently reported. |
| Gelli et al., 2019 | A school meals program implemented at scale in Ghana increases Height-for-Age during midchildhood in girls and in children from poor households: A cluster randomized trial | 2019 | 2. Quantitative randomized controlled trials | Green | Robust design, study well explained. Methods of analysis could be presented more clearly. |
| Van der Hoeven et al., 2015 | Effect of African leafy vegetables on the micronutrient status of mildly deficient farm-school children in South Africa: A randomized controlled study | 2015 | 2. Quantitative randomized controlled trials | Amber | Small sample size, with a possibly high risk of sample contamination since randomization was done at the individual level and since children ate meals in the same classroom. |
| Abizari et al., 2021 | Free senior high school lunch contributes to dietary quality of nonresidential students in Ghana | 2021 | 3. Quantitative non-randomized | Amber | Confounders reported and explanations of the beneficiaries are included, but finding could be reported more clearly. |
| Desalegn et al., 2022 | Effect of school feeding program on the anthropometric and haemoglobin status of school children in Sidama region, Southern Ethiopia: a prospective study | 2022 | 3. Quantitative non-randomized | Amber | Results are clearly presented, however there is an absence of randomization and differences between intervention and control groups at baseline. |
| Neervoort et al., 2013 | Effect of a school feeding programme on nutritional status and anaemia in an urban slum: A preliminary evaluation in Kenya | 2013 | 3. Quantitative non-randomized | Red | Poorly reported results. The study uses a convenience sample, design is not pre-post and it is hard to determine effect even with the control group, as the intervention and control are not the same in terms of sociodemographic and nutritional status, intervention is school feeding plus others, making it difficult to extrapolate to just school feeding; data collected for control and intervention was at different times; age may not be accurate. |
| Oyela et al., 2023 | Influence of home‑grown school feeding on nutritional status of schoolchildren: Findings from South‑West Nigeria | 2023 | 3. Quantitative non-randomized | Amber | Paper is well presented, but the statistical tests are not clear. There is a high level of stunting at baseline, making it difficult to compare the programmes’ success or lack thereof. Note that estimates may be due to different confounding variables associated with children starting school. |
| Faber et al., 2013 | Is the school food environment conducive to healthy eating in poorly resourced South African schools? | 2013 | 4. Quantitative descriptive (Non comparative study | Red | Attempt to get views from many stakeholders, with broad representation in South Africa of poor schools across different geographical contexts, but the sample size is small for food handlers and qualitative research methods seem more appropriate. As the research tools are not available it is hard to see how the questions were phrased and interpreted. |
