# Supplementary material for: How do publicly procured school meals programmes in sub-Saharan Africa improve nutritional outcomes for children and adolescents: a mixed-methods systematic review
Source: Public Health Nutr. 2024 Oct 18;27(1):e213. doi: 10.1017/S1368980024001939 (PMC11604325; doi:10.1017/S1368980024001939)
Supplement: Liguori et al. supplementary material 4 — Liguori et al. supplementary material [file S1368980024001939sup004.docx]

**Supplementary File 5:** A selection of illustrative quotes on facilitators and barriers to implemention of school meals programmes in sub-Saharan Africa

| **Facilitators** |  |
| --- | --- |
| **Wholesale and trading** |  |
| Mensah, 2019 | *“Our price and the local market price, there is difference. Let assume if we are buying a bag of maize at GHS60.0046, we can decide to attach something small like GHS 5.00, in order to help us sell [the grains] faster”* [Food caterer] P.497 |
| **Processing and distribution** |  |
| Khama, 2022 | *“My role is focused mainly at the national level as you know but it is still narrowed down to regions, service providers, to schools as well. So, as from my side, I am mainly involved in ensuring that the SFP is improved from how it used to be in the past, to better the way it is managed and run, to prevent losses and better equip school feeding, implementers to play a better role, to make it more efficient and to be able to track down the progress that we were making with the aims of SFP"* P.124 |
| **Food preparation** |  |
| Daitai et al., 2018 | *“We pack food in the store room upon arrival and make sure that our store room is also clean. We also clean our kitchen and all cooking utensils every day after serving food to the learners”* [Food handler] P.68  *“As a way of guaranteeing hygienic conditions, the school established the kitchen far away from harmful potentials which can contaminate the food”* [Programme coordinator] P.72 |
| Banda, 2017 | *“My mother is one of the people who were certified at the hospital to ensure food hygiene as she cooks school meals”* [Student] P.60 |
| **Distribution to students** |  |
| Fernandes et al., 2017 | *“We also serve them our food to vary the food they eat because sometimes they can come and eat rice in the school here so when they get back home we prepare [meals] to vary the food and make it balanced”* [Caregiver, Northern Region] P.17 *“The truth is that because we don’t have money, the small [amount] that we have is what we cook at home for all our school children… if they come and are not satisfied at school, we eat together but if they are satisfied, we enjoy our home meal alone”* [Caregiver, Northern Region] P.17 |
| **Students** |  |
| Dei, 2014 | *“I become strong when I eat the food”* [Student] P.72 |
| Desleagan et al., 2022b | *“My son is learning in this Faleqa primary school, and most of the time I don’t have anything to give him at home as breakfast. Before the SFP started, my son frequently had missed the class preferring to do work for money/food than attending school”* [Caregiver] P.3 |
| Moepeng | *“Most of us have never tasted traditional food, we only hear about them. However, we are taught about these in our classes. Sometimes when teachers ask us in class, we are unable to answer the questions because we don’t know. If we have these foods as part of our menu, we will have better knowledge and understanding”* [Student] P.22 |
| Sanousi, 2019 | *“Yes, children enjoy the food very well, you can see when they are eating, they shake and move their legs. They really like the food because it is tasty, warm and smells nice and also because it’s a different menu every day” (food handler). AND “The learners enjoy the food. I have a problem with some learners– they’re always crowding the kitchen in the afternoon, looking for leftover food”* [School Principal] P.62 |
| **Community involvement** |  |
| Banda, 2017 | *“Food is served on daily basis. As parents we interchange and give one another shifts according to villages and cooks who have been certified by medical personnel to handle pupils food within school peripheral”* [Parent] P.63 |
| Hamupembe, 2016 | *“I am very happy with their service. The fact that these people have been volunteering themselves to help the school it shows commitment towards the education of their children”* [School employee] P.67 |
| Sibanda, 2012 | *“Continuous involvement of the community in the school activities leading to a good synergistic relationship between the school and the community”* [Caregiver] P.82 |
| **Barriers** |  |
| **Food production** |  |
| Mensah & Karriem, 2021 | *“Produce more by increasing the size of my plot and the number of crops cultivated”* [Farmer, female] P.10 |
| **Wholesale & trading** |  |
| **Decentralised** |  |
| Khama, 2022 | *“We have tried to decentralise the programme. We are busy, but we can already see challenges from the first stages of decentralisation. Regions are unable to pay timely, they are not able to monitor its effectively, and they will rely on the Head Office sometimes to knock on them to say hello, how far is the progress in terms of deliveries?”* P.17 |
| **Local procurement** |  |
| Daitai et al., 2018 | *“The programme is not supporting local farming since the middleman are purchasing their products from outside the community”* [Supervisor at circuit level, female, responsible for several schools] P.84  *“I can supply any kind of product required by the schools throughout the year but currently there is no reason to fully participate in farming since we lack reliable market opportunities”* [Farmer] P.85 |
| Mensah & Karriem, 2021 | *“There was one lady who supplied us with cabbages. I don’t know maybe it’s because of the drought, she has not come back to supply us again and so we buy from the big shops”* P.7 |
| **Transportation & Storage** |  |
| **Irregular food deliveries** |  |
| Banda, 2017 | *“All the 68 schools including community schools were on school feeding apart from one school which is located very far from DEBS office and has impassable roads. This makes delivery of food rations very difficult”* [District officer] P.61  *“The problem with this programme is consistence of food supply to schools, you know….learners get used to eating at school, a sudden cut in the supply of food discourages them…. and thus absenteeism becomes high…..learners cannot predict when they would have the next meal”* [Headteacher] P.75 |
| Daitai et al., 2018 | *“Sometimes we do not prepare food because the suppliers always fail to give us food in time and as a result learners will not eat”* [Food handler]. P.91 |
| Molotja, 2019 | *“The main challenge with the SFPs is non-delivery of food by the suppliers”* [Programme Officer] P.97 |
| **Late food deliveries** |  |
| Dei, 2014 | *“I sometimes have to use my personal money to buy vegetables in order for learners to have a complete meal”* [School Principal] P.81 |
| Mafugu, 2021 | *“The challenge of the principal is when the food is not delivered on time. The timetable has to change to accommodate the feeding time”* P.4 |
| Sanousi, 2019 | *“There was a delay in the delivery and when we reported the problem, the food was immediately delivered. There was an incident where, for the whole week, there was no food and the school bought bread from its own resources. So the principal had to buy food from school resources, and this was caused by the delay in delivery.[...], “They told us the truck was on the way, but it never came. They said the truck had been delayed but we didn’t understand. So the children were given just bread, bread, bread for whole week and there was nothing to add to the bread”* [Food handler, female] P.63 |
| **Quality of food delivered (spoiled)** |  |
| Desalegn et al., 2022 | *“ . . . We had a problem of cereal quality at the time of delivery. We received the food through the farmer’s union and they delivered some already spoiled cereals bags. For example, in our school, out of 68⋅5 quintals of maize, 20 quintals of maize were already spoiled at the time of delivery. We appealed the problem to the district education office but so far we haven’t got any response”* [School director] P.4 |
| **Infrastructure** |  |
| Daitai et al., 2018 | *“The school did not receive any funds for storage facilities and we are not able to raise money for the construction of a storeroom, but the hygienic demands of the programme compelled the school to utilise one of the classroom as a store room*” [Coordinator of school meals programme] P.70. |
| **Processing and distribution** |  |
| **School gardens** |  |
| Daitai et al., 2018 | *“We used to produce good cabbages, carrots, onions and tomatoes supplementing the programme at this school while we got some remuneration from the suppliers, but they later failed to pay us and our garden started to go down”* [Supervisor at circuit level, female, responsible for several schools] P.86 |
| Sanousi, 2019 | *“There is a food garden, but it does not produce sufficient food, it might be sufficient for a particular meal that is cooked for that day, because it’s a small garden.”* P.64 |
| **Food preparation** |  |
| **Delayed payments** |  |
| Mafugu, 2021 | *“Sometimes the service provider will say: ‘The department has not paid me. I can’t provide students exactly as they should be provided’”* P.4 |
| Mensah, 2019 | *"While the grain banks are helping the school feeding programme, the delayed payment [by government] is not making the grain banks grow fast…imagine as we speak, I owe the grain banks for about eight months. This is because government is yet to pay me for my services"* (Food caterer) P.497  *“Payment of caterers is not regular at all. You know if [the caterer] feeds the children for a whole term, they should be paid. That’s the contractual agreement. But sometimes caterers are not paid for two terms yet they are expected to prepare meals for the children. How will they work? … Payment on the part of government has been very poor. This affects the programme because the women complain bitterly”* P.497 |
| Yendaw & Davour, 2015 | *“Look my brother, the government does not release money to the school on time, so the matron cannot do her best to provide good food. You see, the food sometimes is not that fine and even the quantity is sometimes small but what for do, the matron cannot use her own money to cook for the children. We just have to pray that the government increases the support”* [40 y, male, Head Teacher] P. 384 |
| **Unpaid and untrained food handlers** |  |
| Ellis, 2012 | *"It has been a challenge for most schools to arrange volunteer cooks. Often five hours of work per day is involved, which is difficult for anyone to offer without appropriate reward. The schools that have succeeded in arranging cooks did so at a cost, in terms of providing bags of food or using part of the school development fund as payment to cooks"* P.59 |
| Hamupembe, 2016 | *“The manual should include the financial aspects of school feeding, especially the payment of cooks”* P.75 |
| Khama, 2022 | *“The compensation of our cooks is a problem here, and a serious challenge. They are demanding some compensation. I support their claim of compensation because they are coming from poor backgrounds. Look, instead of the cooks going to markets to sell what they have in order to get money to sustain themselves, they will be coming to school for two weeks where they would not be paid”* P.170 |
| Mafugu, 2021 | *“There is a challenge for workshop attendance. Sometimes the venue is not accessible. Some principals do not transport food handlers. Some food handlers do not attend due to logistical problems. This, in turn, results in food handlers lacking cooking techniques while service providers lack business skills”* P.4 |
| **Infrastructure** |  |
| Hamupembe, 2016 | *“We need a proper kitchen, currently we are using a storeroom in the school hall as a cooking facility”* P.64 |
| Khama, 2022 | *“When you look at our kitchen, it is really not good. During this rainy season, once it rains heavily, our learners will not eat at all, it will be difficult to kindle the fire’’* P.135  *“Our kitchen has a temporal structure, when it rains, those sinks are sometimes blown out by wind and the porridge become affected by dust”* P.136 |
| **Lack of nutrition knowledge** |  |
| Ellis, 2012 | *“Almost all the children interviewed, let alone the cooks and teachers, were not aware of what is in the porridge, and mostly thought it to be maize meal with milk and sugar, as is the tradition. There is little understanding of the nutritional value (and real cost) of the maize blend"* P.46 |
| **Programme requirements** |  |
| Langsford, 2018 | *“I noticed several towers of porridge packets in the storeroom and later found out that the school does not supply breakfast in the mornings even though it should. When I asked about the bags it was confirmed that these were porridge bags but no explanation about why they were stacked there was offered”* P.40 |
| **Tools for measurement and consistency** |  |
| Fernandes et al., 2016 | *“The use of the handy measures has also ensured that the amount of food served is the right quantity the child requires”* [District Desk Officer from Mpohor, Western Region, Ghana. P.580 |
| **Distribution to students** |  |
| **Workload** |  |
| Banda, 2017 | *“Some parents do not cooperate at this school…., teachers themselves end up cooking for the learners. Teachers often time cook in the night as they supervise learners during prep since this is a weekly boarding, then we finalise the following day. Basically the teacher on duty takes the lead in cooking and supervising if done with pupils”* [Teacher] P.75 |
| Khama, 2022 | *"Whilst the NSFP attracts learners to school, it also brings additional responsibilities on top of the teachers teaching loads"* P.122  *"I am not willing to be trained, because let me say, it is too much for me. I am a class teacher at the end of the term I have to complete those end term reports. I have a lot to do. This is additional work on top of my work".* P.128 |
| **Quantity and quality** |  |
| Daitai et al., 2018 | *“There should be strong efforts applied by the government on the amount of food to be given to each learner. We are experiencing a gradual decrease of the quantity of supplies year after year and currently it gives me a mental headache calculating the quantity to be served per day such that the food might be enough for the whole month”* [Coordinator of school meals programme]. P.79 |
| Molotja, 2019 | *“We do not get full, they serve little food”* [Learner] P.81  *“We get sore tummies when we eat soup and samp at school”* [Learner] P.81 |
| Sanousi, 2019 | *“The problem is that sometimes they supply us with cheaper food which is not of a good quality, like rice or soya, it is not nice. Our learners tell us that the food does not taste nice and they eat less. The learners come to us and complain about food, they know exactly when it tastes different. They ask, ‘What’s wrong with the food today? This showed that they supplied us with the wrong quality. Sometimes they also don’t supply us with fresh fruits and vegetables”* [Food handler] P.52 |
| **Time constraints** |  |
| Khama, 2022 | *“Time is very much affected, we experience some delays and some class cutting because our learners need time to line-up, time to receive that hot porridge, time to eat, time to wash their hands and their plates. This implies that some learners will miss the first period from break because they will still be eating”* P.173 |
| **Lack of utensils/cutlery** |  |
| Ellis, 2012 | *"Children come with inadequate ‘plates’ such as plastic lunch boxes, or none at all, and try to share with those that do have something. Pot lids can be commandeered. The cleanliness of these assorted receptacles also leaves much to be desired […] the school development fund has purchased bowls and spoons, which are rinsed by the children after eating but washed and retained by the school "* P.46 |
| Hamupembe, 2016 | *"We do not have enough utensils, few pots, no spoons and few plates. We need enough for the kids”* P.72 |
| Khama, 2022 | *“The challenges we face are related to kitchen utensils that we use for the SFP. Like spoons, our learners are using hands to eat instead of spoons and our school cannot afford to buy spoons for our learners. The plates, dishes and pots are few, we only have two pots, when learners are many; some will not get anything”* P.166 |
| **Meal diversity** |  |
| Hamupembe, 2016 | *“We want other food, or fruits to supplement the current meal”* P.61 |
| Desalegn et al., 2022b | *“Including some fruit and vegetables to the school food would increase the benefit that the students get from the school food”* [School director]. P.4 |
| Moepeng, 2016 | *“In school we are encouraged to grow vegetables; however we don’t get to eat any of these; instead the school sells them. This is not fair as we contribute to producing food that we can eat right here in school. We want a more balanced diet which includes vegetables and fruit”* P.41 |
| **Monitoring (record keeping)** |  |
| Molotja, 2019 | *“Some NSNP educators do not keep proper records; they do not file all documents that they are supposed to file”* [NSNP Officer] P.67 |
| **Food safety** |  |
| Desalegn et al., 2022b | *“We don’t have water in our school compound, even in the near distance to the school. For these reasons we required each student to bring a bottle of water from his/her home to attend the school meal of the day. We don’t have budget to buy water daily to cook food for thousands of students”* P.4 |
| **Students** |  |
| **Preferences** |  |
| Daitai, 2018 | *“We are not used to eat soya and even milk together with pap… this kind of meal sometimes force us to abscond from the food”* [Student] P.89 |
| Ellis, 2012 | *“However, the learners are not very keen on the taste of the porridge and consistently ask that more sugar be added. Some schools have responded to the request by adding sugar to the maize blend at the expense of the school development fund. Children can also be seen adding sugar or powdered sweeteners that they have brought from home"* P.45 |
| Mafugu, 2021 | *“Students complained about soya mince and rice on Tuesdays and Thursdays. There were no alternative meals for learners who did not like the standard meals”* P.4 |
| Molotja, 2019 | *"School meals give us energy, nourish our bodies, make us strong and they are nutritious"* [Student] *P.79* |
| **Stigma** |  |
| Khama, 2022 | *“They say that ‘you are eating porridge because you are poor’’. So, those learners become demotivated, especially those who are in grades 6 and 7 - their emotions are so sensitive. Though would be hungry, they would stop eating the porridge because they would be afraid that their friends will laugh at them”* P.176 |
| Sibanda, 2012 | *“It would be better if the programme was implemented in all the schools in the country so that it helps all the children to have a better future.” They noted that the current situation whereby some schools and some learners are in the school supplementary feeding programme presence a form of stigma to those eating the food. Though acknowledging the magnitude of the resources required, the participants suggested that the school feeding programme need to cover all the school and all the learners so that they all eat comfortably without stigma”* P.84 |
| **Perceptions** |  |
| Khama, 2022 | *“…Some of the learners do not want to eat the porridge fearing that they will have a running stomach or diarrhoea”* P.136 |
| **Community involvement** |  |
| **Perceptions** |  |
| Khama, 2022 | *“The impact is just little, I noticed that learners’ meals are not diversified. In fact, learners are taught in school that it is not appropriate for them to eat the same type of food, they should rather eat a variety of food to have a balanced diet”* P.138 |
| Sichala, 2020 | *“The lean dietary content of the district’s HSFP menu was not enough to address the nutritional deficiencies of school aged children of the district”* P.61 |
| **Infrastructure Support** |  |
| **Coordination** |  |
| Khama, 2022 | *“I think training needs to be conducted at all levels, all different stakeholders involved need to be capacitated.… even at Head Office, let me say, you have some senior officials, they do not understand the importance of SFP, so, I understand that they too, need to receive training or being given a background so that they understand what the programme is all about - especially those who are involved in the disbursement of funds, they should understand why it is important for us to fund this programme. In addition, those that are involved in the procurement need training to understand why it is necessary for them to timely advertise, adjudicate and award these tenders so that there is no disruption in the food supply”* P.127 |
| **Eligibility** |  |
| Sanousi, 2019 | *“I do think that our school should be reclassified as quintile 3, because there were times school fees were not paid regularly. If we are in quintile 3, we would get more resources to help our poor children. Quintile 3 will be highly subsidised and we will get more attention from the government. We are classified as the same schools in Rondebosch, Newlands and other rich areas; these children are definitely not the same in terms of family background”* [Teacher] P.53 |
| **Monitoring and evaluation** |  |
| Banda, 2017 | *“We are not monitored by officers from DEBS office on school feeding. They just concentrate on other aspects of the system… they don’t even mention ‘food’ when these standard officers come for routine monitoring. The problem with DEBS office is that they just rely on monthly reports we send but don’t physically come to visit and see how schools are operating in the area of school feeding”* [Head teacher] P.76 |
| Khama, 2022 | *“You know what? I am worried about those people who are monitoring this programme here in the Zambezi Region. They do not come to see the situation on the ground, and we usually see the company that supplies food. We do not know whether we have bosses or what. We do not know them, they do not visit schools. Maybe if they visit our schools, we might have an opportunity to ask questions”* P.173 |
| Molotja, 2019 | *“We are five NSNP officers responsible for many schools in the five school circuits. These schools are scattered far from each other. We are supposed to visit schools on a daily basis, but we cannot because we share one vehicle among the five of us. Each of us can only visit two schools per week”* [NSNP Officer] P.66 |
