# Supplementary material for: How do publicly procured school meals programmes in sub-Saharan Africa improve nutritional outcomes for children and adolescents: a mixed-methods systematic review
Source: Public Health Nutr. 2024 Oct 18;27(1):e213. doi: 10.1017/S1368980024001939 (PMC11604325; doi:10.1017/S1368980024001939)
Supplement: Liguori et al. supplementary material 5 — Liguori et al. supplementary material [file S1368980024001939sup005.docx]

**Supplementary file 2: Data Extraction Form**

| Initials of reviewer |
| --- |
| Title of article |
| First author (surname only) |
| Year of publication |
| Inclusion criteria (all 4 are required to proceed with full data extraction) |
| Type of publication |
| Single country (yes/no), if yes write the name  (e.g., No OR Kenya) |
| Multiple countries (yes/no), if yes list them all (e.g., No OR South Africa, Kenya, Mozambique) |
| Does the country have a national school feeding programme |
| If yes, what is the name of the programme |
| Context: Is this study conducted with schools/students/etc. that participate in a national school feeding programme |
| If reported, what type of catering model is used? |
| Context: Geographic location of data collection (e.g., the city of Accra)  Note: this is to see if many studies in the same zones, not to make extrapolations on representation |
| Area of data collection |
| Objective (stated aim of study) |
| Study design |
| Type of study |
| Data collection method used |
| Please explain further if different methods were used with different sample populations |
| Type of school |
| Number of schools included (sample size) |
| Sampling of schools |
| Number of participants (sample size) |
| Sampling of students |
| Sample characteristics (participants) |
| Student age range (please note if not reported) |
| Other participants age range (e.g., food vendors) (please note if not reported) |
| Type of intervention |
| Description of intervention |
| Who provided the interventions? (e.g. teachers, volunteers, etc) |
| Duration of intervention, if reported |
| Duration of evaluation, if reported |
| Cost of intervention (cost per beneficiary if reported) |
| Were any theoretical frameworks used to develop the intervention? |
| Results: Outcome(s) measured |
| Evidence to detail each outcome (include page numbers) |
| Confounders reported/considered? |
| Does your study also have qualitative data to analyse? |
| Data Collection Method Used |
| Please explain further if different methods were used with different sample populations  (e.g., food vendors were individually interviewed and parents participated in focus group discussions) |
| Type of school |
| Number of schools included (sample size) |
| Sampling of schools |
| Number of participants (sample size) |
| Sampling of participants |
| Sample characteristics (participants) |
| Student age range (please note if not reported) |
| Other participants age range (e.g., food vendors) (please note if not reported) |
| Outcome(s) measured |
| Evidence to support each outcome selected (use quotation marks, page # and participant attributes if possible) |
| Barriers (or not reported) |
| Quotes on Barriers (include quotation marks, page # and participant attributes if possible) |
| Facilitators (or not reported) |
| Quotes on Facilitators (use quotation marks, page # and participant attributes if possible) |
| Was food consumption recorded? |
| If yes, what tools were used to record food consumption? |
| Their stated recommendations (from text) |
| Policy-specific Recommendations (from text) |
| Personal notes/reflections on the article |
| References to screen for inclusion (copy and paste relevant references) |
| Date of extraction |
